# The influence of herbivory and weather on the vital rates of two closely related cactus species

**DOI:** 10.1002/ece3.3232

**Published:** 2017-07-31

**Authors:** Kristen E. Sauby, John Kilmer, Mary C. Christman, Robert D. Holt, Travis D. Marsico

**Affiliations:** ^1^ Department of Biology University of Florida Gainesville FL USA; ^2^ Department of Biological Sciences Arkansas State University Jonesboro AR USA; ^3^ MCC Statistical Consulting LLC Departments of Biology and Statistics University of Florida Gainesville FL USA; ^4^ Present address: Department of Biology Missouri Southern State University Joplin MO USA

**Keywords:** *Cactoblastis cactorum*, herbivory, *Opuntia*, plant‐insect interactions, precipitation, temperature

## Abstract

Herbivory has long been recognized as a significant driver of plant population dynamics, yet its effects along environmental gradients are unclear. Understanding how weather modulates plant–insect interactions can be particularly important for predicting the consequences of exotic insect invasions, and an explicit consideration of weather may help explain why the impact can vary greatly across space and time. We surveyed two native prickly pear cactus species (genus *Opuntia*) in the Florida panhandle, USA, and their specialist insect herbivores (the invasive South American cactus moth, *Cactoblastis cactorum*, and three native insect species) for five years across six sites. We used generalized linear mixed models to assess the impact of herbivory and weather on plant relative growth rate (RGR) and sexual reproduction, and we used Fisher's exact test to estimate the impact of herbivory on survival. Weather variables (precipitation and temperature) were consistently significant predictors of vital rate variation for both cactus species, in contrast to the limited and varied impacts of insect herbivory. Weather only significantly influenced the impact of herbivory on *Opuntia humifusa* fruit production. The relationships of RGR and fruit production with precipitation suggest that precipitation serves as a cue in determining the trade‐off in the allocation of resources to growth or fruit production. The presence of the native bug explained vital rate variation for both cactus species, whereas the invasive moth explained variation only for *O*. *stricta*. Despite the inconsistent effect of herbivory across vital rates and cactus species, almost half of *O*. *stricta* plants declined in size, and the invasive insect negatively affected RGR and fruit production. Given that fruit production was strongly size‐dependent, this suggests that *O*. *stricta* populations at the locations surveyed are transitioning to a size distribution of predominantly smaller sizes and with reduced sexual reproduction potential.

## 1 INTRODUCTION

Herbivory has long been recognized as a significant driver of plant population dynamics (e.g., Crawley, 1989; Harper, 1977). Because insect herbivores and their plant hosts are likely sensitive to climate, their interactions may shift as patterns of temperature and rainfall change due to global climate change (Stocker et al., 2014). Weather not only has direct effects on plant populations (e.g., on phenology, growth and mortality rates, Cleland et al., 2007), but it also can indirectly affect plant vital rates (*i.e.,* recruitment, death, survival, growth, and reproductive rates) by shifting the outcomes of plant–herbivore interactions (Jamieson et al., 2012), leading to nonlinear effects of insects on plant performance (Amarasekare, 2015). The interplay of herbivory and climatic variation is likely to be complex, and we lack consensus across empirical studies on the effects of herbivory given variation in climatic conditions (Maron et al., 2014).

Understanding how weather modulates plant–insect interactions can be particularly important for predicting the consequences of insect pest outbreaks and/or exotic insect invasions (e.g., Haavik et al., 2015; Mwalusepo et al., 2015). While invasive invertebrates generally reduce plant fitness (Cameron et al., 2016), their impacts can vary greatly across space (Latzka et al., 2016; Reichard et al., 2015) and time (Kulhanek et al., 2011; Strayer et al., 2006); the consideration of climate effects may help explain this variation.

Complicating matters is the fact that plant population dynamics and interactions are likely influenced by weather in ways other than via changes in mean climate conditions. The effects of mean environmental conditions can be magnified or reversed by environmental variance (Cuddington & Hastings, 2016; Lawson et al., 2015), and the variability of an environmental factor can be as or more important than its mean (Goldstein & Suding, 2014; Nagy et al., 2013). Interactions between temperature and precipitation complicate the task of predicting herbivore effects upon plants, which may explain why such interaction effects remain infrequently considered in population models (Ehrlén et al., 2016).

**Figure 1:**
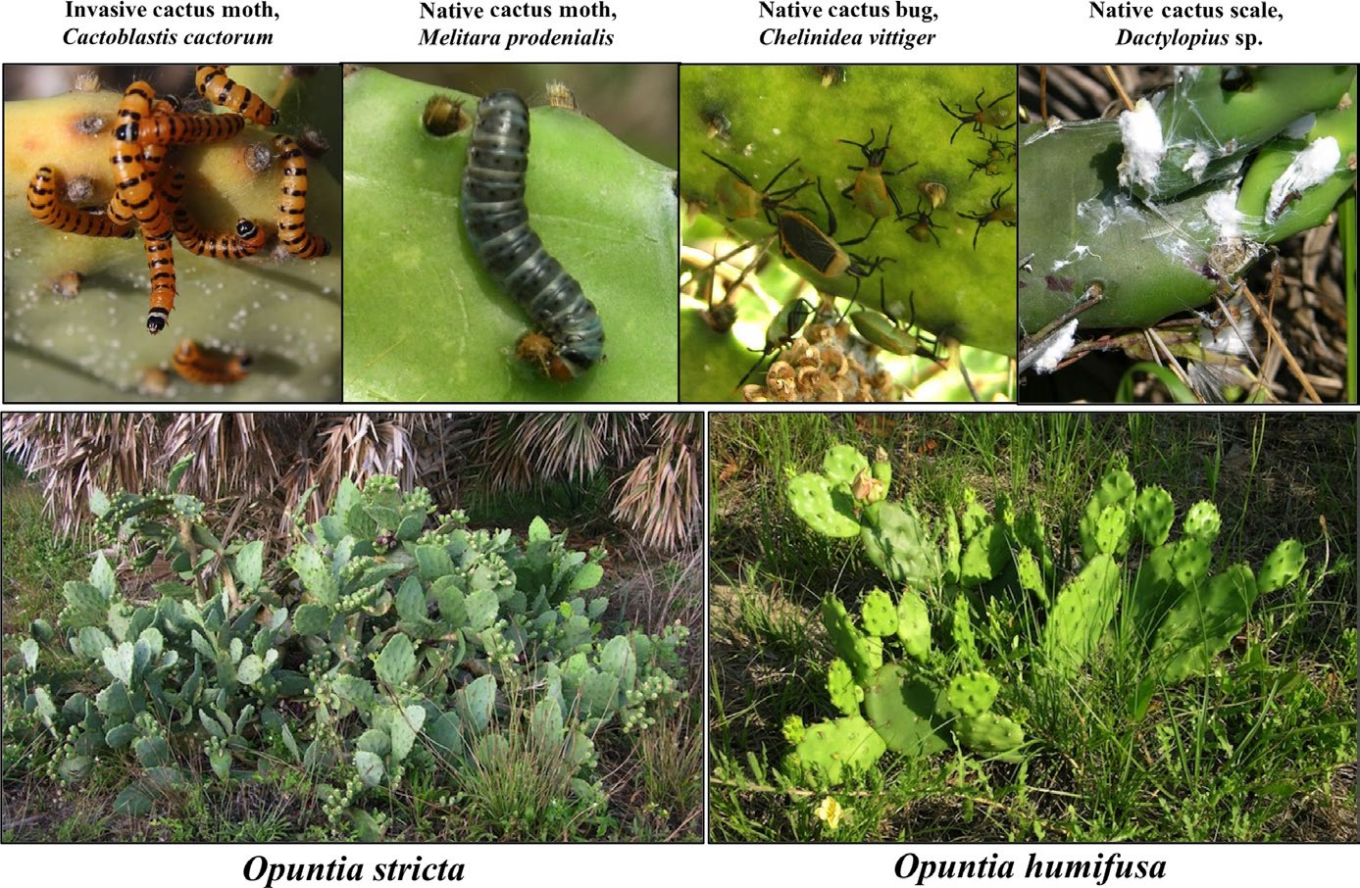
The two cactus and four insect species sampled across the Florida panhandle

We investigated the impact of invasive and native insect herbivory within the context of climate using as our study system two closely related native prickly pear cactus species (genus *Opuntia*) in the Florida panhandle, USA. Four insect species found in this region, which feed exclusively on *Opuntia*, include the invasive South American cactus moth, *Cactoblastis cactorum* (Berg) (Lepidoptera: Pyralidae), and three native insect herbivores: the moth *Melitara prodenialis* Walker (Lepidoptera: Pyralidae), the bug *Chelinidea vittiger* McAtee (Hemiptera: Coreidae), and the scale insect *Dactylopius* species (Hemiptera: Dactylopiidae) (Figure 1). The invasive moth was historically used for biological control of invasive, non‐native *Opuntia* populations elsewhere in the world (e.g., Australia; Dodd, 1940), but has now become invasive in the southeastern USA where *Opuntia* are native (Marsico et al., 2011; Zimmermann et al., 2000). First detected in the Florida Keys in 1989 (Dickel, 1991), the invasive moth has since spread as far west as Louisiana, despite management activities aimed at control (Rose, 2009). While evidence from Australia suggests that this moth negatively and strongly impacts cacti (Dodd, 1940), studies in Florida and elsewhere have shown mixed results with respect to the impact of the invasive moth on cactus vital rates (Hoffmann et al., 1998; Jezorek et al., 2012), possibly because these prior studies have not accounted for environmental conditions (e.g., temperature and precipitation).

We used a five‐year observational study to assess the relative importance of herbivory and weather to cactus growth, sexual reproduction, and survival. We addressed the following questions: (1) Does the presence of insect herbivores negatively affect cactus annual relative growth rate (RGR), sexual reproduction, and survival?; (2) If so, do these effects vary depending on whether the insect species is native or invasive?; (3) Do effects of insect interactions on cactus RGR and sexual reproduction vary depending on weather conditions?; and (4) Are the effects of herbivory and weather qualitatively consistent across the two cactus species?

We first hypothesized that the invasive moth would have a greater negative impact on cactus vital rates compared to the native insects, which have presumably shared a long evolutionary history with these cactus species. Experimental data suggest that the cacti have evolved adaptive counter‐strategies to herbivory by the native moth but not the invasive moth (Woodard et al., 2012). We also hypothesized that the impact of herbivory on vital rates would be diminished during periods of high temperatures, as *Opuntia* may be more heat tolerant than the insects (Nobel & De la Barrera, 2003; Rodríguez‐Castañeda, 2013). We also hypothesized that the impact of invasive moth and native scale herbivory on vital rates would decrease with increased precipitation, as these insect species have been found to be detrimentally affected by heavy rainfall (Mann, 1969; Robertson & Hoffmann, 1989). Moreover, herbivores can reduce the high drought tolerance of *Opuntia*, leading to reduced cactus survival and growth (Hosking & Deighton, 1980; Nobel, 2002). We hypothesized that a negative interaction between temperature and precipitation (the induction of drought effects at high temperatures) would increase the effect of herbivory on plant vital rates. Alternatively, as a null hypothesis, weather could affect vital rates independent of the effects of herbivory. For sexual reproduction, we hypothesized that weather seasonality was important due to the seasonal nature of flower production (Gimeno & Vilà, 2002; Godínez‐Álvarez et al., 2003) and fruit maturation (K. Sauby, unpublished data; Christine Miller, personal communication; Reyes‐Agüero et al., 2006), and that weather variation either in the fall/winter or spring/summer of the previous year would influence fruit production in a given year.

To address our questions and hypotheses, we formulated generalized linear mixed models (GLMMs) to explain variation in cactus RGR and sexual reproduction as functions of insect herbivore presence and/or weather variables. We formulated our models so that each vital rate could be explained by (1) the presence of insects alone; (2) weather alone (precipitation [P] and/or temperature [T]); (3) a combination of insect presence and weather (P and/or T); (4) interactions among an insect species and weather (P or T); (5) interactions among P and T; (6) pooled native insect presence; and/or (7) plant size at survey t (for RGR models)/during fecundity year z (for sexual reproduction models). We ranked competing models using the conditional Akaike Information Criterion (cAIC, Vaida & Blanchard, 2005; Müller et al., 2013) to identify which sets of factors best explained variation in observed RGR and sexual reproduction. We also tested whether cactus survival varied significantly with insect presence.

## 2 METHODS

### 2.1 Study system

*Opuntia humifusa* (Raf.) Raf. var. *humifusa* (hereafter referred to as *O. humifusa*) is distributed throughout the eastern USA, from Florida north to Michigan and west to Texas. *Opuntia stricta* (Haw.) Haw. is limited to the southeastern USA, from coastal Texas to South Carolina, and the Caribbean (Benson, 1982); it is listed as “Threatened” in Florida (FDACS, 2015). Compared to *O*. *stricta*, which can grow as a large shrub up to 2 meters (m) tall, *O. humifusa* usually only reaches 30–60 centimeters (cm) in height (Figure 1, Benson, 1982).

The invasive moth and native moth, bug, and scale insect have been observed feeding on both *O. humifusa* and *O*. *stricta* in Florida and are widespread throughout the state (Figure 1, Sauby, 2009). The invasive moth arrived in the Florida panhandle by 2002 (Hight et al., 2002) and is found primarily along the coast as far north and west as South Carolina (Hight et al., 2002) and Louisiana (Rose, 2009), respectively. The native moth is found across the southeastern USA and shares similar life history traits with the invasive moth (Mann, 1969; Stephens et al., 2012). The larvae of both moth species feed by boring into cactus cladodes (the flat units into which *Opuntia* stems are segmented), leaving excrement and/or hollowed cladodes as evidence of their presence. The cactus bug and scale insect are native to and widespread throughout North America (Mann, 1969). Feeding damage by the native bug and scale insect can lead to the loss of young cladodes and fruit; feeding by the native bug appears as circular, bleached marks (Dodd, 1940). Both moths and the native bug undergo two to three generations per year in Florida (Hight & Carpenter, 2009; Legaspi et al., 2008; Mead & Herring, 1974), while the native scale insect undergoes four to five (Mann, 1969).

### 2.2 Sampling data collection

**Figure 2:**
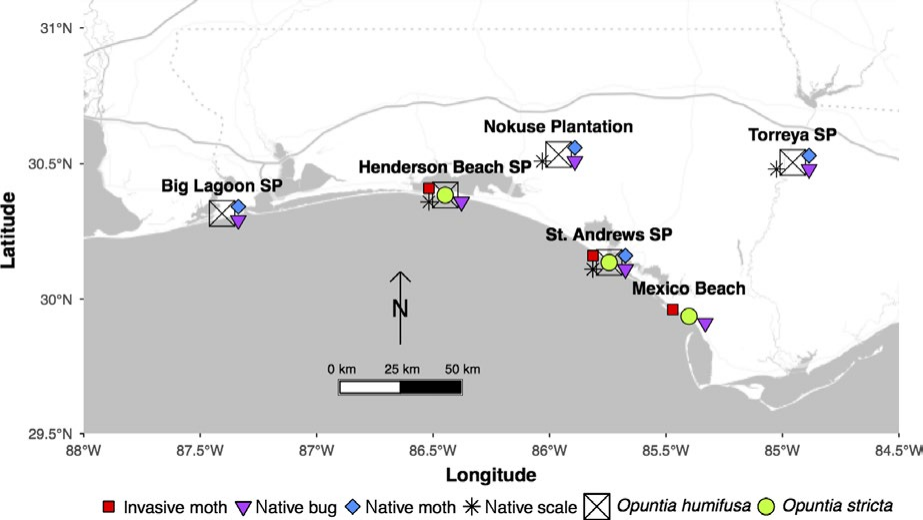
Sampling locations in the Florida panhandle and the cactus and insect species detected there. “State Park” is abbreviated as “SP”

Cacti were surveyed 12 times at six locations over a five‐year period: four times in 2009 (January, April, July, and October) and once every May and between November and January from January 2010 to January 2014 (Figure 2). In January 2009, plants were marked and surveyed along one to two transects per location, and 15–20 cacti of each species, if present, were surveyed (median number per species per location marked during the first survey: 18; total marked *O. humifusa* and *O*. *stricta* across all locations: 106 and 54, respectively). For most transects, cacti were sampled at evenly spaced intervals within a 4 m wide transect; transect lengths differed among locations due to variation in the spatial extent and density of cactus populations (see details below). If no cacti were located within the given interval, the next closest cactus within the transect was sampled.

Patches of *O. humifusa* plants were relatively large at Nokuse Plantation (NP) and Torreya State Park (TSP), and cacti at each site were sampled at 5‐m intervals along a 100‐m transect. At Big Lagoon State Park (BLSP), patches of *O. humifusa* plants were smaller, and cacti were sampled at 5‐m intervals along two 50‐m transects. At Henderson Beach State Park (HBSP), *O. humifusa* plants were selected at shorter intervals along a 50 m transect. At St. Andrews State Park (SASP), neighboring (if possible) *O*. *stricta* and *O. humifusa* plants were sampled at approximately 5‐m intervals along a 100‐m nonlinear transect. Because patches of *O*. *stricta* were smaller at HBSP and Mexico Beach (MB), the first 15–20 individuals encountered along a transect were sampled.

Marked cacti were surveyed by photographing each plant and recording size (the number of cladodes) and fruit abundance. Insects were recorded as present if individuals were directly observed, with a few exceptions. The cactus bug was recorded as present if eggs, nymphs, and/or adults were observed on the cactus. We also recorded it as present if new, distinct plant damage since the last visit could be attributed specifically to that species, regardless of whether individuals were observed. The presence of the cactus moth species was based on the detection of eggsticks (masses of eggs laid in chains resembling spines) and/or larvae, which can be identified easily to species (Dodd, 1940; Mann, 1969; Stephens et al., 2012) and detected most weeks of the year in the Florida panhandle (Legaspi et al., 2008, 2009). In cases where moth damage had been inflicted since the previous survey, but eggsticks and larvae were not observed (e.g., larvae may have been too deeply burrowed into cladodes), moth damage was recorded and assigned to a moth species by (1) considering if the species was present at the location, (2) inspecting nearby nonmarked cacti, and (3) examining location and plant and insect survey records for the five‐year study. For five of the six locations, only one moth species was detected during the study (Figure 2), and in these instances, moth damage was assigned to that species. At the sixth location, SASP, both moth species were detected, and multiple plants were host to both species during the course of the study. At that location, almost all instances of moth damage were identified to species based on visual identification of moth individuals present at the time. Only six instances of moth damage at SASP (and the only six during the entire study period across all six sites) could not be identified to species.

For each cactus that died or could not be relocated, a new cactus nearby and within the transect was added to the set of marked plants so that up to 30 cacti were surveyed per species per location (median number of cacti per species per location: 18.5). We excluded surveys of eight cacti at HBSP from May 2011 onward from analyses of RGR and sexual reproduction because of biomass removal due to invasive moth management by the United States Department of Agriculture (USDA; personal communication, Arthur Stiles, Research and Collecting Permit Coordinator, District 1 Parks, Florida State Parks). We also excluded these particular cacti from our survival analyses.

We created datasets for each of the RGR, sexual reproduction, and survival analyses. The RGR dataset contained observations from each of the 12 surveys. The sexual reproduction dataset summarized fruit presence and maximum (max.) fruit abundance, max. plant size, and insect presence for each fecundity year; each fecundity year started on the first day of spring in year *z* and ended on the last day of winter in year *z *+ 1 (e.g., fecundity year 1 summarized data collected 20 March 2009 through 19 March 2010). The survival dataset was restricted to cacti marked at the beginning of the study and observed during each survey until death or the end of the study; this summarized whether each cactus had ever been infested by any of the four insect species and whether it had survived the five‐year study.

### 2.3 Weather data

We acquired daily max. and minimum (min.) temperature (Celsius [°C]) and daily total precipitation (cm) data from the National Oceanic and Atmosphere Administration National Climatic Data Center (Menne et al., 2015) for the March 2008–January 2014 period. We used data from the weather stations closest to each of our sampling locations and filled in missing values with data from the next closest station (number of stations used per sampling location: 2–7; range of distances between weather stations and sampling locations: 2.87–64.68 km). Weather stations located more than 30 km from a cactus sampling location were used only to fill in missing values, so that the average station distance per daily weather value was 16.1 km. Only 3.7% of daily weather values came from weather stations more than 30 km away from a cactus sampling location.

From these weather data, we derived 16 variables that we hypothesized would capture the average of and variation in weather conditions. For the RGR datasets, we calculated each weather variable using data from the date of survey t to the day before survey *t *+ 1. For sexual reproduction, we calculated each weather variable separately for the first and second six months of the year prior to the start of the “fecundity year” (e.g., spring–summer 2008 and fall 2008–winter 2009 weather data for fecundity year 1 survey data [spring 2009–winter 2010]).

Precipitation variables included daily precipitation total (mean and standard deviation [SD]), percentage of days with rain, the number of consecutive days with rain (mean, max., and SD), and the number of consecutive days without rain (mean, max., and SD). Temperature variables included metrics of heat accumulation (daily max. temperature [mean and SD] and average degree day [for details see Appendix S1; UCDIPM, 2015]) and variables that measured the degree of exposure to freezing temperatures (the percentage of days with freezing temperatures and the number of consecutive days with freezing temperatures [mean, max., and SD]).

### 2.4 Statistical analysis

#### 2.4.1 Weather

We reduced the number of weather variables to be included in our regression models by performing principal components analyses (PCAs; McCune et al., 2002) on our weather data using SAS software (PROC FACTOR method = principal, SAS/STAT version 13.2, SAS Institute Inc., Cary, NC, USA; code available upon request). To ease interpretability and put variables on a similar scale, we first standardized the weather variables (Gelman, 2008; Schielzeth, 2010; “rescale” function in the R “arm” package, Gelman & Su, 2015; R Core Team 2015), which were then included in the PCAs. We performed separate PCAs for each of the two cactus species because the weather stations used differed (due to differences in the sets of sampling locations at which the species occurred; Figure 2). Using PCA, we reduced the number of weather variables to one or two independent composite variables for each of the weather types (precipitation and temperature; Appendix S1, Tables S1–S2). For each PCA, we retained axes that had eigenvalues >1; if two axes were retained, we rotated them to improve interpretability (PROC FACTOR, SAS/STAT version 13.2).

For the *O. humifusa* sexual reproduction analysis, instead of performing a PCA on spring/summer temperature data, we included standardized spring/summer mean daily max. temperature and standardized spring/summer average degree day directly in the models. A PCA including the other spring/summer temperature variables was not appropriate given that daily max. temperature could not be transformed to normality and that the remaining variables described aspects of freezing weather. Finally, because of limited survey data for *O*. *stricta*, we did not include weather data in our *O*. *stricta* sexual reproduction analysis.

#### 2.4.2 Relative growth rate

We modeled RGR using linear mixed models (LMMs). We used the number of cladodes as our measure of plant size and included standardized plant size at survey *t*, $C_{t,\mathrm{st}}$, in our candidate models. We calculated annual RGR as the yearly rate of production (or loss) of cladodes, per existing cladode, using the formula ${\mathrm{RG}R}_{\mathrm{ij}}=365\times \left(C_{ij,t+1}-C_{ij,t}\right)/\left(d\times C_{ij,t}\right)$, where $C_{ij,t}$ is the number of cladodes observed for individual *j* of species *i* at survey *t* and *d* is the number of days between surveys t and t+1 (as in Paine et al., 2012).

**Figure 3:**
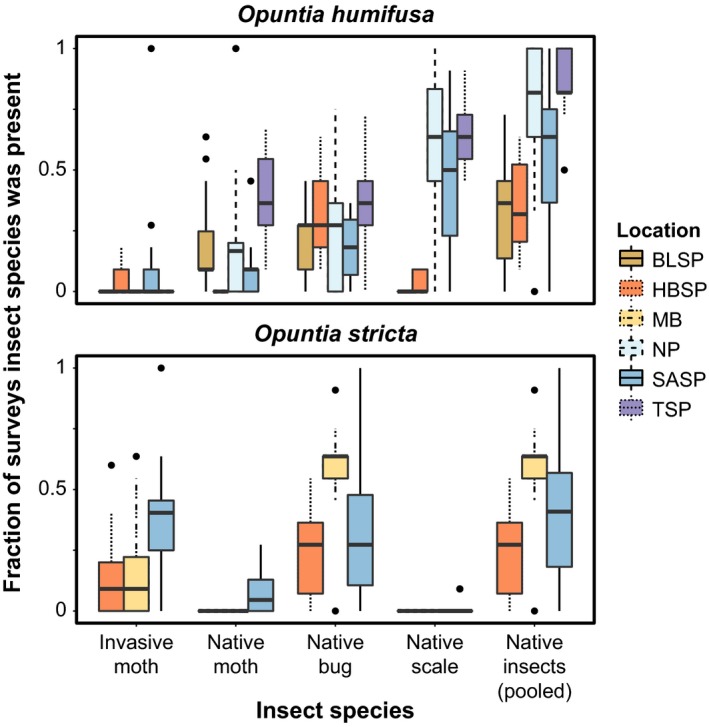
The fraction of the total number of surveys during the 5‐year study period in which each insect species was found on individuals of the two cactus species (*i.e*., the number of surveys the insects was observed, divided by the total number of surveys). The upper and lower lines of the boxes correspond to the 25th and 75th percentiles while the horizontal line inside of the box corresponds to the 50th percentile. The vertical lines (“whiskers”) extend to 1.5 times the distance between the first and third quartiles. The points indicate values that lie beyond the whiskers. Site abbreviations: Big Lagoon State Park (BLSP), Henderson Beach State Park (HBSP), Mexico Beach (MB), Nokuse Plantation (NP), St. Andrews State Park (SASP), and Torreya State Park (TSP)

For each cactus species, the full candidate models included the fixed‐effect variables P1 and P2 (the factor loading scores from the first and second axes of the precipitation PCA, respectively), T1 (the factor loading scores from the first axis of the temperature PCA), and insect presence/absence data from survey *t*. For *O. humifusa*, we included native moth, bug, and scale insect presence/absence; the invasive moth was rarely observed (Figure 3). For *O*. *stricta* models, we included presence/absence of the invasive moth and native bug; the native moth and scale insect were excluded due to rarity (Figure 3). For both cactus species, we also considered the pooled presence/absence of the three native insect species as a fixed‐effect variable.

For *O*. *stricta* models, we included location × year as a random effect because observations at a location tended to be correlated within a year with possible differences among years. For *O. humifusa* models, we included plant identity, because the repeated observations among individual plants tended to be correlated, and location  ×  year as random effects (Appendix S2, Table S1 and S3).

We fit candidate LMMs by varying the fixed effects from the full models and considering interactions among fixed effects (insect species × P, insect species × T, P × T) to evaluate our hypotheses about factors that could affect RGR. For each cactus species, we ranked the candidate models using the conditional Akaike Information Criterion (cAIC; Vaida & Blanchard, 2005; Müller et al., 2013). Using a conservative approach, we used the formula cAIC = ‐2 conditional Log‐Likelihood $+2\left(f+r\right)$, where *f* and *r* are the number of fixed‐ and random‐effect parameters, respectively. For each candidate model, we also calculated ΔcAIC, the cAIC difference between a candidate model and the candidate model with the lowest cAIC. We favored the candidate model with the lowest cAIC, except in cases where a candidate model had ΔcAIC ≤2 and fewer parameters than the candidate model with the lowest cAIC (following Arnold, 2010). In those instances, we favored the most parsimonious candidate model (Appendix S2, Table S2 and S4).

Unfortunately, some of our marked cacti at HBSP were targeted by the USDA for invasive moth management, which involved the removal of a large amount of each plant's biomass. We compared the effects of USDA management and invasive moth infestation on RGR by restricting our dataset to only observations of *O*. *stricta* at HBSP during the surveys immediately before and after USDA management was observed. We categorized cacti by “Management” (whether cacti had ever been managed by the USDA during our study period) and observations by “Time” (whether the survey occurred before or after USDA management). To test whether RGR values declined because of USDA management and/or invasive moth infestation, we fit a LMM containing Management  ×  Time, Management ×  Invasive Moth, and Time  ×  Invasive Moth interactions as fixed effects with plant identity as a random effect (Proc GLIMMIX, SAS/STAT version 13.2).

#### 2.4.3 Sexual reproduction

We used zero‐inflated GLMMs to explain variation in fruit presence and abundance in fecundity year *z*. The full set of fixed effects of both zero and count components of the *O. humifusa* model included the variables $C_{z,\mathrm{st}}$, $P1_{\mathrm{SS}}$, $P2_{\mathrm{SS}}$, $P1_{\mathrm{FW}}$, $P2_{\mathrm{FW}}$, $\mathrm{Mean}\;\mathrm{Max}.\;\mathrm{Daily}\;\mathrm{Temp}._{\mathrm{SS}}$, $\mathrm{Mean}\;\mathrm{Degree}\;\mathrm{Da}y_{\mathrm{SS}}$, $T1_{\mathrm{FW}}$, and native moth, bug, and scale insect presence/absence; SS and FW represent spring/summer and fall/winter weather data, respectively. In contrast to *O. humifusa*, many *O*. *stricta* plants were never observed with fruit (35 of the 52 that were alive for at least two consecutive surveys) and only a small number varied in terms of fruit presence during the study (10/52). This restricted the number of predictors that we could fit to the data, preventing us from testing all of our hypotheses. Thus, for the *O*. *stricta* model we limited the fixed effects of the zero and count models to $C_{z,\mathrm{st}}$ and invasive moth and native bug presence/absence.

For each of the two cactus species, we first fit a zero‐inflated generalized linear model (Binomial and Poisson families for the zero and count models, respectively) according to the above sets of fixed effects and retained in the model only those fixed effects that were statistically significant (α=0.05, Proc GENMOD, SAS/STAT version 13.2). We then fit zero‐inflated GLMMs with the subset of significant fixed effects (Proc NLMIXED, SAS/STAT version 13.2, Appendix S3, Tables S1 and S3). For *O. humifusa*, we included plant identity as a random effect in the abundance component of the model. For *O*. *stricta*, we included fecundity year as a random effect in the zero component and location ×  fecundity year in the abundance component of the model. Finally, for *O. humifusa*, we considered the inclusion of interactions (insect species  ×  P, insect species  ×  T, P  ×  T) among the restricted set of fixed effects and performed model selection using cAIC (Appendix S3, Tables S2).

#### 2.4.4 Survival

Due to the small number of plants that died during the study period, we analyzed survival as a function of insect infestation using Fisher's exact test (PROC FREQ Fisher option, SAS/STAT version 13.2). We tested whether mortality was higher given infestation by a particular insect during the 5‐year study period. For *O. humifusa*, we tested the effects of the three native insect species on survival, and for *O*. *stricta,* we tested the effects of the invasive moth and native bug.

Data are available from the Dryad Digital Repository: https://doi.org/10.5061/dryad.p4q26 (Sauby et al., 2017).

## 3 RESULTS

*Opuntia humifusa* and *O*. *stricta* were found at five and three of the locations, respectively. Unlike *O. humifusa*,* O*. *stricta* was restricted to coastal sites (Figure 2). The three native insects were relatively common on *O. humifusa* (Figure 3), in contrast to the invasive moth which was found on only 10% (11/106) of *O. humifusa* plants. At our inland sites, NP and TSP, as well as a coastal site, SASP, many *O. humifusa* individuals were repeatedly infested by the native scale and sometimes simultaneously infested by the native moth. In contrast, at BLSP and HBSP many *O. humifusa* individuals were less frequently infested over the course of the study, and at HBSP many cacti were repeatedly infested by only the native bug and at BLSP by only the native bug and moth. Most *O*. *stricta* individuals were infested at least once by the invasive moth and native bug during the study (Figure 3).

### 3.1 Weather

For all PCAs, at least 74% of the variation in the nine precipitation and 79% of the six temperature variables was explained by the first two axes (Appendix S1, Tables S1–S2). For both cactus species and vital rates, the signs and magnitudes of the factor loading scores also were relatively consistent. The P1 axes were strongly correlated with rainy weather and the P2 axes were strongly correlated with dry weather. The T1 axes were positively correlated with freezing temperatures. For the RGR datasets, the T1 axes were also negatively correlated with mean max. temperature and mean degree day and positively correlated with the SD of max. temperature and the max. number of consecutive days with freezing temperatures. For the *O. humifusa* fall/winter fecundity dataset, the T2 axis was strongly and positive correlated with mean max. temperature (Appendix S1, Tables S1–S2).

### 3.2 Relative growth rate

Many *O*. *stricta* plants were smaller at the end of the study than at the beginning (48%; 21 of the 44 that were alive at the end of the study), compared to 25% that were larger. The percentage of plants that declined in size also varied by location (HBSP: 12%, MB: 47%, and SASP: 92%). In contrast, most *O. humifusa* plants were larger at the end of the study (57%; 43 of 76 that were alive at the end of the study) and the percentage varied by location (BLSP: 53%, HBSP: 61%, NP: 25%, TSP: 85%, and SASP: 31%). However, 38% declined in size.

The RGR of both cactus species was explained in part by standardized plant size, $C_{t,\mathrm{st}}$, and weather. The RGR of both cactus species was negatively associated with $C_{t,\mathrm{st}}$ (Table 1), and the effect for *O. humifusa* was more than double that of *O*. *stricta*. In terms of weather, the RGR of both cactus species was positively associated with dry weather (RGR was positively associated with P2; Table 1). Additionally, *O. humifusa* RGR was negatively associated with rainfall (it was negatively associated with P1) and metrics of heat accumulation and positively associated with metrics of exposure to freezing and with the SD of max. temperature (it was positively associated with T1; Table 1). For *O*. *stricta*, a positive interaction between P1 and T1 explained variation in RGR, suggesting that the impacts of precipitation and temperature on that species are interdependent; such an interaction by contrast was not included in the best model for *O. humifusa*.

**Table 1:** For each cactus species, vital rate, and dataset, parameter estimates (including 95% confidence intervals in brackets) are given for the fixed effects included in the best models (out of the sets evaluated) explaining *Opuntia humifusa* and *Opuntia stricta* RGR and sexual reproduction. Presence/absence is abbreviated by a “+/−.” A “−” indicates that the variable was not included in the best model, and a “NA” indicates that the variable was not considered in any of the candidate models for that cactus species and vital rate

Vital rate	RGR	RGR	Fruit absence	Fruit abundance	Fruit absence	Fruit abundance
Species	*O. humifusa*	*O*. *stricta*	*O. humifusa*	*O. humifusa*	*O*. *stricta*	*O*. *stricta*
Intercept	0.189 [−0.01, 0.388]	0.361 [0.153, 0.57]	0.2 [−0.36, 0.75]	0.91 [0.64, 1.18]	3.37 [1.39, 5.35]	0.15 [−0.3, 0.61]
*C*_t,st_	−0.632 [−0.838, −0.426]	−0.25 [−0.439, −0.06]	−1.16 [−2.12, −0.2]	2.57 [2.3, 2.83]	−4.46 [−6.98, −1.95]	5.16 [4.86, 5.47]
Native Bug +/−	‐	−0.201 [−0.398, −0.004]	−0.6 [−1.29, 0.09]	0.05 [−0.08, 0.18]	–	−0.93 [−1.27, −0.59]
Native Moth +/−	–	–	–	–	–	–
Native Scale +/−	–	–	–	–	–	–
Native Insects +/−	–	–	–	–	–	–
Invasive Moth +/−	–	−0.4 [−0.627, −0.172]	–	–	–	−0.39 [−0.5, −0.27]
Precipitation	P1 = −0.172 [−0.267, −0.077], P2 = 0.153 [0.075, 0.232]	P1 = −0.02 [−0.135, 0.096], P2 = 0.187 [0.083, 0.291]	P1 (Spring/Summer) = −0.63 [−0.98, −0.28]	P1 (Fall/Winter) = 0.13 [0.08, 0.18]	NA	NA
Temperature	T1 = 0.196 [0.112, 0.278]	T1 = 0.348 [0.239, 0.457]	Mean Max. Temp (Spring/Summer) = −2.21 [−3.05, −1.37]	Mean Degree Day (Spring/Summer) = −1.49 [−1.79, −1.19], Mean Max. Temp (Spring/Summer) = 0.55 [0.31, 0.8], T1 (Fall/Winter) = 0.22 [0.14, 0.3]	NA	NA
Precipitation × Temperature	–	P1 × T1 = 0.201 [0.059, 0.343]	–	–	NA	NA
Insect × Weather	–	–	–	Native Bug × Mean Degree Day (Spring/Summer) = 0.68 [0.39, 0.96]	NA	NA
Random Effect	Location × Year, Plant ID	Location × Year	–	Plant ID	Fecundity Year	Location × Fecundity Year

**Figure 4:**
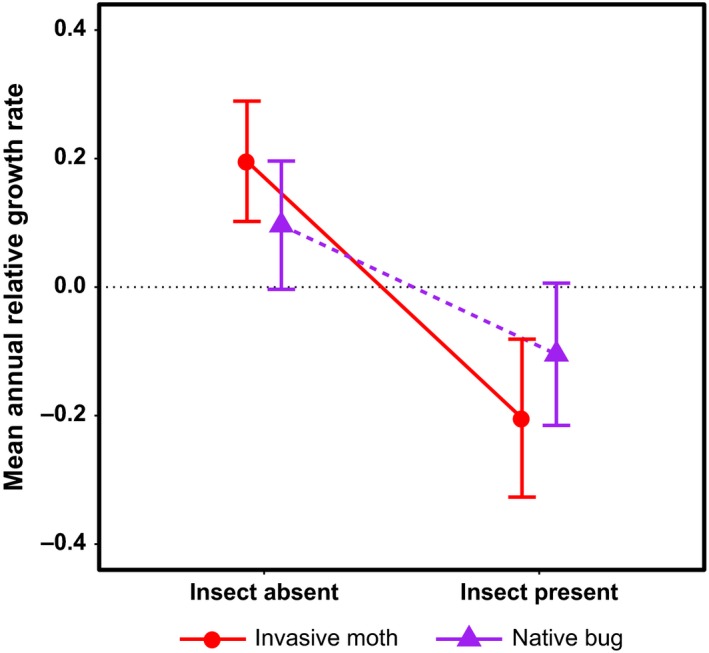
Predicted *Opuntia stricta* annual RGR in the presence and absence of the invasive moth and native bug, based on the best model (Table 1) and made by fixing the other variables in the model, including P1, P2, and T1, at their averages. The distance from the points to the end of the error bars is one standard error

However, the effect of insect presence was not a consistent predictor of RGR. *Opuntia stricta* RGR was negatively affected by the presence of both the invasive moth and the native bug (Figure 4). In contrast, the best model among those tested for *O.  humifusa* did not include any insect variables (Table 1).

At HBSP, an unplanned manipulation in the form of USDA management of the invasive moth was introduced into our study of *O*. *stricta*. Among *O*. *stricta* plants there, USDA management unsurprisingly caused a significant decline in RGR (management ×  time: *p *= .0058). While some *O*. *stricta* plants were found infested by the invasive moth at HBSP during this time period, the moth did not significantly affect RGR at this site (invasive moth, *p *= .2476; time  ×  invasive moth, *p *= .7867; management  ×  invasive moth, *p *= .1879).

### 3.3 Sexual reproduction

**Figure 5:**
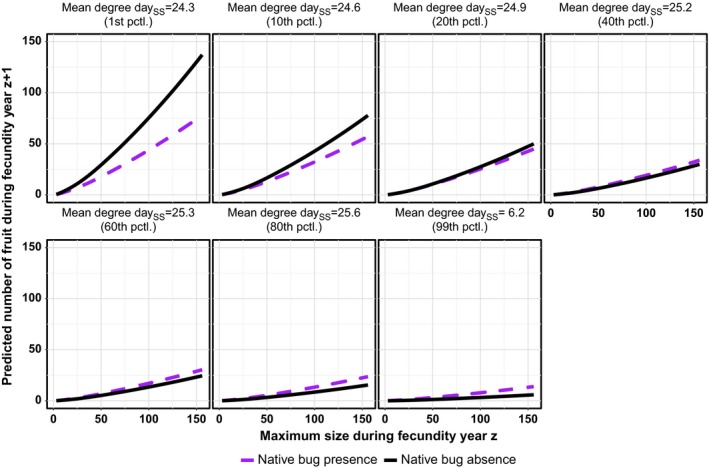
The predicted effect of native bug presence/absence on the relationship between *Opuntia humifusa* maximum plant size during year z and number of fruit produced in year z+1, at different percentiles (pctl.) of spring/summer mean degree day. Predictions are based on the best model (Table 1) and made by fixing the values of other variables in the model at their averages

**Figure 6:**
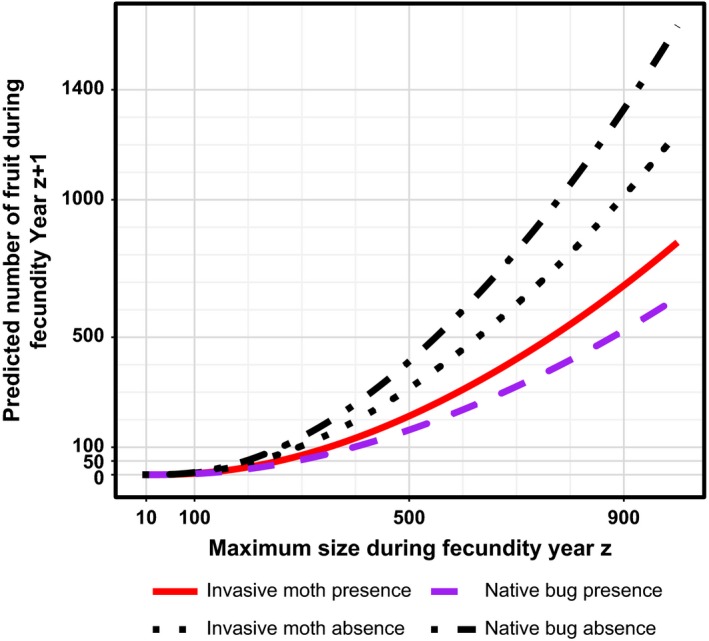
The effect of the invasive moth and native bug presence/absence on the relationship between *Opuntia stricta* maximum plant size during year z and number of fruit produced in year z+1. Predictions are based on the best model (Table 1)

As with RGR, the presence and abundance of fruit was positively associated with standardized plant size, $C_{z,\mathrm{st}}$, for both cactus species (Table 1; Figures 5, 6). Herbivory was also an important predictor for both cactus species, although the signs of the effects and the identities of the influential insects differed by cactus species. For *O. humifusa*, the relationship between the number of fruit and the presence of the native bug switched from being negatively to positively associated as spring/summer mean degree day increased (Figure 5). In contrast, the presence of the native bug and invasive moth were negatively associated with *O*. *stricta* fruit abundance, and the impact of the native bug was more than twice that of the invasive moth (Figure 6).

Weather was also an important driver of *O. humifusa* sexual reproduction. (Because so few *O*. *stricta* plants produced fruit, no weather variables were included in the models of *O*. *stricta* sexual reproduction.) Fruit presence and abundance were both positively affected by spring/summer mean max. temperature. Fruit abundance was also negatively associated with spring/summer mean degree day (Figure 5) and positively associated with freezing temperatures in the fall and winter (*i.e*., positively associated with the fall/winter T1). Additionally, both fruit presence and abundance were positively associated with rainfall: fruit presence was positively associated with rainfall in the spring and summer of the previous year and fruit abundance with rainfall in the previous fall and winter (*i.e*., positively associated with the spring/summer and fall/winter P1 axes, respectively).

### 3.4 Survival

For *O. humifusa* and *O*. *stricta*, the overall mortality rates were 16.5% (15 of 91 plants followed since the first survey) and 12.2% (5/41), respectively. *Opuntia humifusa* survival was positively associated with native bug presence: 89% of plants infested at least once during the study period survived compared to 40% of plants never infested (Fisher's exact test: *p *= .0011). However, *O. humifusa* survival was not affected by the presence of the other native insects (native moth, Fisher's exact test: *p *= 1; native scale insect, Fisher's exact test: *p *= .075). The survival of *O*. *stricta* was not affected by any insects present (native bug, Fisher's exact test: *p *= 0.12; invasive moth, Fisher's exact test: *p *= 1).

## 4 DISCUSSION

In our study, we found that weather was consistently important to explaining variation in vital rates across cactus species, while the impacts of herbivory were not consistent. Only the native bug and invasive moth were included as variables in our best models explaining variation in RGR and sexual reproduction, and the effects of these species varied from positive to negative. Native bug presence was positively associated with *O. humifusa* survival and negatively with *O*. *stricta* RGR and sexual reproduction (Figures 4 and 6), whereas invasive moth presence was negatively associated with *O*. *stricta* sexual reproduction and RGR (Figures 4 and 6). The effect of native bug presence on *O. humifusa* sexual reproduction also varied from negative to positive as spring/summer mean degree day increased (Figure 5). Our hypothesis that the invasive moth would have a greater negative impact compared to the native insects was supported only for *O*. *stricta* RGR. The native bug in fact had a greater negative impact than the invasive moth on *O*. *stricta* sexual reproduction (Figure 6), and the invasive moth was not included in any other best vital rate models.

Furthermore, we found inconsistent support for our hypotheses about interactions of weather. Only *O*. *stricta* RGR was explained by an interaction among precipitation and temperature. Also, the impact of herbivory varied with weather (mean degree day, in particular) for only *O. humifusa* fruit abundance (Figure 5). None of the best models included interactions between herbivores and precipitation, suggesting that the data do not support the hypothesis that herbivores affect *Opuntia* drought tolerance or that herbivory is reduced with increased precipitation. In contrast, our analyses showed that weather, independent of other predictor variables, was important for most vital rates in which the variables were considered. Weather alone explained variation in *O. humifusa* RGR and, in combination with insect presence, explained variation in *O*. *stricta* RGR and *O. humifusa* sexual reproduction.

### 4.1 Herbivory

The invasive moth was only significantly associated with *O*. *stricta*, the species that has been found to be the most frequently infested by the invasive moth in Florida relative to other cactus species (Sauby et al., 2012). We speculate that the invasive moth's negative effect on *O*. *stricta* could be due to selective pressure the moth has faced to survive while feeding on *O*. *stricta*, given the moth's recent history of colonization around the globe. The moth first rose to fame after its successful control of millions of acres of *O*. *stricta* in Australia in the 1920s and 1930s, following its introduction there from its native range in South America (Dodd, 1940). The moth was then taken from Australia to South Africa to control invasive *Opuntia* (Pettey, 1948). From there, moths were introduced to the Caribbean island of Nevis to control native *Opuntia*, including *O*. *stricta* (Simmonds & Bennett, 1966). The invasive moth then arrived in Florida by 1989 (Dickel, 1991), likely from the Caribbean (Marsico et al., 2011; Pemberton, 1995). Thus, the invasive moths observed in the Florida Panhandle had ancestors that underwent selective pressure over many generations in Australia and the Caribbean to persist on *O*. *stricta*. The invasive moth's negative effects on *O*. *stricta* RGR and fruit abundance that we found in our study could be due to the naiveté of these populations of *O*. *stricta* to the feeding of this introduced specialist herbivore in the southeastern USA (Woodard et al., 2012), which has been present for relatively few cactus generations.

Alternatively, the relative difference in infestation of *O*. *stricta* and *O. humifusa* could be due to species trait differences in average height— *O*. *stricta* is generally taller and may be more readily detected by females of the invasive moth (Sauby et al., 2012). Also, we did not find an effect of the invasive moth on survival because few *O*. *stricta* plants died during the study period, and USDA management inadvertently reduced our sample size. A larger sample size and/or longer study period could provide the power needed to detect a significant, meaningful effect of the invasive moth on survival. Additionally, based on the way we determined plant size and survival, it is difficult for a plant to be completely eliminated by the insect. If whole cladodes or fragments of cladodes that had separated from the original plant survived, the original plant was considered to have survived because individuals genetically identical to the original plant survived. (This change in plant size structure resulting from invasive moth herbivory for *O*. *stricta* can potentially have profound impacts on reproduction and population structure; see additional discussion in Plant Size section below).

In addition to previous research, our study has shown that *O. humifusa*, despite also being naïve to the invasive moth, does not experience the same degree of infestation by it (Jezorek et al., 2012; Sauby, 2009). In our study, we did not include the invasive moth in our candidate vital rate models for *O. humifusa* because the moth was so rarely observed on that species; it was found on *O. humifusa* at only two sites, and only 11 plants (of 106 total) were ever infested by the invasive moth during the study (Figure 3). While two of our locations with *O. humifusa* were inland (TSP and NP, Figure 2) and in areas where the invasive moth has not yet ever been recorded, three of the *O. humifusa* locations (BLSP, HBSP, and SASP; Figure 2) were on the coast and well within the range of invasion. Yet, the invasive moth was never observed at BLSP where *O*. *stricta* was not detected, and it was found feeding on half as many marked *O. humifusa* as *O*. *stricta* plants at HBSP (6/18 and 13/19, respectively) and SASP (5/17 and 14/17, respectively).

The relatively low infestation rates of *O. humifusa* by the invasive moth may be due to the invasive moth not having the same history of association with *O. humifusa* as it does with *O*. *stricta*;* O. humifusa* has not been a significant concern as an invasive plant species outside its native range. Alternatively, the invasive moth may have been present at our sites in the past but is now largely absent; a history of past herbivory by the invasive moth on *O. humifusa* could explain the fact that plants were on average shorter at sites with the invasive moth (6.9 cm in height; HBSP and SASP sites) than at sites where the invasive moth was never observed infesting *O. humifusa* cacti (13.6 cm in height; BLSP, NP, and TSP sites). However, we certainly cannot rule out a range of alternative explanations (e.g., it is due to a history of herbivory by a different insect species and site environmental differences). Moreover, while cacti can be induced to defend against the invasive moth when fed on by the native moth (Woodard et al., 2012), the two moth species co‐occurred at only one site with *O. humifusa* (SASP), and thus, induced defense is unlikely to explain completely the difference in infestation rates of *O*. *stricta* and *O. humifusa*.

In contrast to the invasive moth, native bug presence explained vital rate variation for both cactus species (Figures 4, 5, and 6). This could be due to an ability of the insects to identify and select healthier plants (e.g., Miller et al., 2006). Alternatively, the association may reflect induced defense; evidence suggests that plants damaged by insect herbivory may experience increased fecundity compared to controls (Agrawal, 1999). Induction may be more likely for native specialist herbivore species with which the cacti have presumably coevolved.

### 4.2 Weather

The amount of rainfall in the previous year as indicated by the P1 axis appears to be important to the resource allocation trade‐off. For *O. humifusa*, RGR and fruit abundance had opposite relationships with rainfall. In general, rainfall was negatively associated with RGR. In contrast, rainfall of the previous year was positively associated with sexual reproduction, matching results from another study of *Opuntia* (Bowers, 1996). Thus, greater rainfall may signal to *Opuntia* to allocate more resources to fruit rather than to growth. (Because so few *O*. *stricta* plants produced fruit, no weather variables were included in model selection to explain *O*. *stricta* sexual reproduction, and thus, we could not assess this potential trade‐off for *O*. *stricta*.)

While there is some criticism of the use of rainfall data for analyzing plant growth, given that precipitation is not a direct measure of water availability (Moles et al., 2014), we found that precipitation was important to explain variation for both cactus species and multiple vital rates. Precipitation is likely a strong determinant of soil moisture in the well‐drained, sandy Florida soils (Saha et al., 2008; Weekley et al., 2007) in which *Opuntia* are typically found. Precipitation may also be important to plants at coastal sites because there is an increased risk of saltwater intrusion into groundwater sources during periods of reduced precipitation (Greaver & Sternberg, 2010).

While temperature was consistently important across both cactus species and for the vital rates for which it was considered, the relationships were complex. RGR of both cactus species was negatively related to variables quantifying heat accumulation (T1 axes), while in contrast, *O. humifusa* sexual reproduction was positively related to mean max. temperature of the previous spring and summer, negatively related to mean degree day of the previous spring and summer, and positively related to freezing temperatures of the previous fall and winter.

### 4.3 Plant size

The effect of plant size on RGR for *O. humifusa* was more than double that for *O*. *stricta*, indicating that *O. humifusa* experiences stronger self‐limitation in terms of individual growth, which agrees with our general observation that *O. humifusa* individuals are on average smaller than those of *O*. *stricta* (Sauby, 2009). Additionally, our best models showed that the threshold size for the initiation of fruit production, as well as the number of fruit produced per size unit increase in *O*. *stricta,* was almost twice that of *O. humifusa*. For both cactus species, the importance of plant size to fruit production suggests that if the population stage structure transitions to a predominance of small plants because of insect herbivory or some other cause (e.g., as happened with populations of *O*. *stricta* infested with the invasive moth in South Africa, Hoffmann et al., 1998), populations may experience diminished persistence probabilities or decreased genetic diversity due to a reliance on clonal rather than sexual reproduction. Given that almost half of *O*. *stricta* plants declined in size during the study, and also that the invasive insect negatively affects RGR (Figure 4) and sexual reproduction (Figure 6, Table 1), this type of transition in population stage structure may well be underway at the locations sampled for this study.

### 4.4 Random effects

We accounted for the possibly confounding effects of our sampling design by utilizing the GLMM framework. Plant identity was only warranted for inclusion in *O. humifusa* models, indicating that there may be more individual‐level variation in vital rates in that species compared to *O*. *stricta* (Table 1; Appendix S2, Table S1; Appendix S3, Table S1). There was also significant location‐by‐year variation in RGR for both cactus species and significant location‐by‐fecundity year variation for *O*. *stricta* sexual reproduction (Table 1; Appendix S2, Tables S1 and S3; Appendix S3, Table S3). This suggests that there is year‐specific variation unique to each location that is not accounted for by our set of predictor variables. Fecundity year also explained a significant amount of variation in *O*. *stricta* fruit presence, also suggesting that year‐specific variation is not accounted for by any of the other variables in our models (Table 1).

### 4.5 Caveats

A complicating factor about this system is that the larvae of the invasive and native moths feed inside cladodes, making the insects difficult to count without destructive sampling of entire cacti. To minimize the impact of sampling, we recorded a moth species as present if at least one larva or eggstick was observed. Our data are thus presence/absence, masking potentially important variation in insect abundance among plants. Thus, we are unable to infer the degree, if any, to which the relative effects of the insect species was due to numerical differences in abundance. However, experimental evidence has shown that the cactus response to moth herbivory does vary by moth species, independent of insect abundance (this study and Woodard et al., 2012). In future studies, the impact of insect abundance could be studied through manipulative experiments using insect abundance as a treatment. Alternatively, the fraction of times an individual plant is observed infested by an insect could be used.

There may be indirect environmental effects on vital rates that we were unable to account for here. For example, we do not know whether the effects of the insects on the cacti are mediated by climate, or whether instead the insects mediate how climate affects the cacti. For instance, freezing temperatures may negatively affect insect herbivory, thus having a positive, indirect effect on *O. humifusa* RGR. Also, there may be local adaptation to environmental conditions that reduce the impact of weather or herbivory on vital rates, potentially explaining why we did not see stronger effects of interactions among weather and herbivory. Additionally, daily temperature and precipitation value were measured at weather stations located on average 16.1 km away from cactus sampling locations, and thus may not perfectly capture the weather conditions actually experienced by the cacti in this study.

Finally, our sample size of *O*. *stricta* was inadvertently and unfortunately reduced due to USDA management of several (mostly large) *O*. *stricta* plants at HBSP sometime between November 2010 and May 2011 to attempt to control an invasive moth infestation. We observed in May 2011 that these plants were much smaller than they had been during our November 2010 survey. Despite the aggressive USDA management, however, the invasive moth infestation continued, and these managed plants continued to decline in size through December 2011. Because of this unplanned manipulation, we removed observations of these plants after the management period from our RGR and sexual reproduction analyses and did not include these plants in our survival analyses. Because our study ended in January 2014, we do not know the extent to which these plants have recovered, or whether these management activities impacted invasive moth abundance.

### 4.6 Future directions and conclusions

An important avenue of research in climate change ecology will be to investigate how indirect effects of climate (such as on plant–insect interactions) affect population growth rates; few studies have done so to date (Ehrlén et al., 2016). This elucidation of indirect effects is particularly important because biotic and abiotic effects on plant performance do not necessarily translate to equivalent effects on plant population growth (Dahlgren & Ehrlén, 2009; von Euler et al., 2014; Maron et al., 2010; Urban et al., 2016). Urban et al. (2016) recently noted this as one of several lacunae in the use of climate change models to make accurate ecological forecasts. The inclusion of important biotic and abiotic effects can make population models more realistic (Oppel et al., 2014) and help predict how population growth rates will change with a changing climate (e.g., Dalgleish et al., 2011).

Thus, an understanding of the effects of herbivory and weather on cactus demography in Florida is important, particularly given the spread of the invasive moth and the risk it poses to native cacti, yet measurements of cactus RGR and sexual reproduction at our sites will not provide a complete understanding of these effects. For instance, while a majority of *O*. *stricta* plants declined in size during the study, we do not know whether these declines in size will actually translate to a decline in the population size. Our ultimate goal should be to incorporate vital rate relationships into population models. To do so, additional information about survival, reproduction, and recruitment is needed. Only a very small number of marked plants died over the course of our study, making inference about the relative importance of weather and herbivory for survival difficult. Additionally, we measured only sexual reproduction, but *Opuntia* can also reproduce clonally. For some *Opuntia* populations, cloning may be the dominant form of reproduction (Mandujano et al., 2007; Palleiro et al., 2006). Also, some studies have found that the relative reliance on sexual versus clonal reproduction is influenced by precipitation (Mandujano et al., 2001; Smith et al., 2005). This could have major consequences for the effective population size and genetic diversity of a population, because a heavy reliance on clonal reproduction reduces the adaptive potential of a population (but see Van Drunen et al., 2015). Additionally, recruitment information will indicate whether population‐level biomass losses to insect feeding are replaced in the form of the recruitment of new individuals.

We caution that correlational studies such as what we have presented cannot definitively indicate causality. Nonetheless, the patterns reported here provide useful springboards for future experimental studies. Further studies on the effects of weather could be particularly interesting, given that the importance of weather to vital rates can dwarf that of herbivory. In our study, we saw evidence suggesting that patterns of rainfall and temperature may affect plant resource allocations, yet there are few empirical studies that explore the effect of weather on the scheduling of plant reproductive allocation (Wenk & Falster, 2015). Finally, given the growing recognition of the need to incorporate biological details into projections of climate change impacts (Urban et al., 2016), continued study of this system can shed light on how the relationships of vital rates with weather vary temporally.

## CONFLICT OF INTEREST

None declared.
