# Supplementary material for: The influence of herbivory and weather on the vital rates of two closely related cactus species
Source: Ecol Evol. 2017 Jul 31;7(17):6996–7009. doi: 10.1002/ece3.3232 (PMC5587481; doi:10.1002/ece3.3232)
Supplement: Supplementary file 1 [file ECE3-7-6996-s001.pdf]

Appendix S1: Analysis of Weather Data

for the article:
Sauby KE, Kilmer J, Christman MC, Holt RD, Marsico TD. The influence of herbivory and weather on the vital rates of two closely related cactus species. Ecol Evol. 2017;00:1–14.
https://doi.org/10.1002/ece3.3232

1 Calculation of Mean Degree Day

We included average degree-day to account for the accumulation of thermal energy available for growth and reproduction (Cayton et al. 2015). We calculated degree-day using the sine-wave method (UCD IPM 2015; Baskerville and Emin 1969; Roltsch et al. 1999). We based the lower threshold temperature of *O. stricta* and *O. humifusa* on information available for *O. ficus-indica* (L.) Mill., a well-studied, agricultural species that has a northern range limit relatively similar to that of *O. stricta* (Benson 1982). Two metrics of cellular activity, acid accumulation and nightly net carbon dioxide uptake, occur between 0 °C and 35 °C (Nobel and Hartsock 1984), and cell damage in cladodes and fruit occurs below 0 °C (Nobel and De la Barrera 2003). Thus, we imposed a 0 °C lower temperature threshold but excluded an upper temperature threshold because *Opuntia* are highly heat tolerant and damage to cladode, root, and fruit cells is not known to occur below 55 - 65 °C (Nobel and De la Barrera 2003).

2 Diagnosis of Non-Normality Among Weather Variables

We used quantile-quantile plots with 95% confidence intervals (function "qqPlot" in the "car" package in R; Fox and Weisberg 2011; R Core Team 2015) to diagnose non-normality among derived weather variables; when necessary, these variables were transformed (Tables S1 - S2). Variables that could not be transformed to approximate normality were excluded from the PCAs.

3 Results from Principal Components Analyses

Table S1: Factor loading scores generated by Principal Components Analyses (PCAs) of precipitation data for each cactus species and vital rate. The "P1" and "P2" columns display the factor loading scores from the first and second axes, respectively, of a PCA; each of the axes had eigenvalues > 1. For the *O. humifusa* fecundity analysis, separate PCAs were calculated using precipitation data restricted to the Spring and Summer as well as the Fall and Winter. Numbers in parentheses are the squared factor loading scores, which give the proportion of variance explained. Abbreviations of summary statistics include maximum (Max.) and standard deviation (SD). If variables were transformed prior to the PCA, the transformation is included in brackets next to the factor loading score and squared factor loading score; transformations included the natural logarithm (ln) and square root (sqrt). All axes were rotated orthogonally to improve interpretability. Underlined variables have relatively high scores for that axis (≥ 0.7), and relatively low scores for the other axis (≤ 0.3).

Variable	Summary Statistic	P1	P2	P1	P2	P1 (Spring/ Summer)	P2 (Spring/ Summer)	P1 (Fall/ Winter)	P2 (Fall/ Winter)
Vital Rate		RGR	RGR	RGR	RGR	Fecundity	Fecundity	Fecundity	Fecundity
Species		*O. humifusa*	*O. humifusa*	*O. stricta*	*O. stricta*	*O. humifusa*	*O. humifusa*	*O. humifusa*	*O. humifusa*
Daily Precipitation	Mean	0.77 (0.6)	-0.04 (0)	0.67 (0.46)	-0.18 (0.03)	0.85 (0.73) [ln]	-0.01 (0) [ln]	0.84 (0.7) [ln]	-0.22 (0.05) [ln]
	SD	0.59 (0.35) [ln]	0.12 (0.02) [ln]	0.45 (0.2)	0.04 (0)	0.71 (0.51) [sqrt]	0.01 (0) [sqrt]	0.72 (0.52) [ln]	0.13 (0.02) [ln]
Percentage of Days with Rain		0.7 (0.49)	-0.63 (0.39)	0.67 (0.45)	-0.66 (0.44)	0.74 (0.55)	-0.58 (0.33)	0.73 (0.53) [ln]	-0.6 (0.36) [ln]
Number of Consecutive Days with Rain	Mean	0.92 (0.85) [ln]	0.01 (0) [ln]	0.91 (0.83) [ln]	-0.08 (0.01) [ln]	0.86 (0.74)	0 (0)	0.95 (0.89) [ln]	0 (0) [ln]
	Max.	0.85 (0.72) [ln]	-0.15 (0.02) [ln]	0.86 (0.74)	-0.07 (0)	0.86 (0.75) [ln]	-0.32 (0.1) [ln]	0.76 (0.58) [ln]	-0.12 (0.02) [ln]
	SD	0.93 (0.87) [ln]	-0.06 (0) [ln]	0.93 (0.86)	0.01 (0)	0.91 (0.83) [ln]	-0.08 (0.01) [ln]	0.92 (0.85) [ln]	0.07 (0) [ln]
Number of Consecutive Days without Rain	Mean	-0.26 (0.07)	0.86 (0.74)	-0.34 (0.12)	0.86 (0.73)	-0.38 (0.15) [ln]	0.77 (0.59) [ln]	-0.4 (0.16)	0.8 (0.64)
	Max.	0.15 (0.02) [ln]	0.86 (0.74) [ln]	0.13 (0.02) [sqrt]	0.91 (0.84) [sqrt]	0.1 (0.01)	0.88 (0.77)	0.14 (0.02)	0.88 (0.77)
	SD	0.08 (0.01) [ln]	0.95 (0.9) [ln]	0.05 (0) [ln]	0.96 (0.92) [ln]	0 (0) [ln]	0.97 (0.94) [ln]	0.08 (0.01)	0.97 (0.95)
Eigenvalue		4.22	2.56	4.20	2.45	4.76	2.25	4.53	2.55
Cumulative Proportion of Variance Explained		0.47	0.75	0.47	0.74	0.53	0.78	0.50	0.79

Table S2: Factor loading scores generated by PCA of temperature data for each cactus species and vital rate. The "T1" and "T2" columns are the factor loading scores from the first and second axes, respectively, of a PCA; each of the axes had eigenvalues > 1. For the RGR analyses, only the first axes had eigenvalues > 1; for the fecundity analysis the first two axes had eigenvalues > 1. Variable transformations included the natural logarithm (ln), square root (sqrt), and power function ($x^{1.25}$ [^1.25] and $x^{0.75}$ [^0.75]). The "Rotated" row indicates which axes were rotated orthogonally to improve interpretability; PCAs of the RGR datasets were not rotated because only one axis per dataset was retained.

Variable	Summary Statistic	T1	T1	T1 (Fall/Winter)	T2 (Fall/Winter)
Vital Rate		RGR	RGR	Fecundity	Fecundity
Species		*O. humifusa*	*O. stricta*	*O. humifusa*	*O. humifusa*
Maximum Temperature	Mean	-0.87 (0.75)	-0.85 (0.72)	-0.08 (0.01)	0.99 (0.97)
	SD	0.75 (0.57)	0.72 (0.52)	0.7 (0.49)	-0.62 (0.39)
Mean Degree Day		-0.93 (0.86) [ln]	-0.91 (0.82) [sqrt]	-0.81 (0.65)	0.51 (0.26)
Percentage of Days with Freezing Temperatures		0.94 (0.88) [sqrt]	0.95 (0.9) [sqrt]	0.96 (0.92)	0 (0)
Number of Consecutive Days with Freezing Temperatures	Mean		0.93 (0.86) [^1.25]	0.92 (0.86)	-0.21 (0.04)
	Max.	0.93 (0.87) [sqrt]	0.95 (0.9) [sqrt]	0.81 (0.66) [sqrt]	-0.49 (0.24) [sqrt]
	SD			0.73 (0.53) [^0.75]	-0.61 (0.37) [^0.75]
Eigenvalue		3.94	4.73	5.35	1.03
Cumulative Proportion of Variance Explained		0.79	0.79	0.76	0.91
Rotated		No	No	Yes	Yes

References

Baskerville GL, Emin P (1969) Rapid estimation of heat accumulation from maximum and minimum temperatures. Ecology 50:514–517. doi: 10.2307/1933912

Benson L (1982) The cacti of the United States and Canada. Stanford University Press, Stanford, California, USA

Cayton HL, Haddad NM, Gross K et al (2015) Do growing degree days predict phenology across butterfly species? Ecology 96:1473–1479. doi: 10.1890/15-0131.1

Fox J, Weisberg S (2011) An R companion to applied regression, 2nd edn. Sage, Thousand Oaks, CA

Nobel PS, De la Barrera E (2003) Tolerances and acclimation to low and high temperatures for cladodes, fruits and roots of a widely cultivated cactus, *Opuntia ficus-indica*. New Phytol 157:271–279.

Nobel PS, Hartsock TL (1984) Physiological responses of *Opuntia ficus-indica* to growth temperature. Physiol Plant 60:98–105.

R Core Team (2015) R: A language and environment for statistical computing. R Foundation for Statistical Computing, Vienna, Austria

Roltsch WJ, Zalom FG, Strawn AJ et al (1999) Evaluation of several degree-day estimation methods in California climates. Int J Biometeorol 42:169–176. doi: 10.1007/s004840050101

University of California Davis Statewide Integrated Pest Management Program (UCDIPM) (2015) How to manage pests: Run models and calculate degree-days. http://www.ipm.ucdavis.edu/WEATHER/ddretrievetext.html.
