# Supplementary material for: The influence of herbivory and weather on the vital rates of two closely related cactus species
Source: Ecol Evol. 2017 Jul 31;7(17):6996–7009. doi: 10.1002/ece3.3232 (PMC5587481; doi:10.1002/ece3.3232)
Supplement: Supplementary file 2 [file ECE3-7-6996-s002.pdf]

Appendix S2: Selection of Models Predicting Relative Growth Rate (RGR)

for the article:
Sauby KE, Kilmer J, Christman MC, Holt RD, Marsico TD. The influence of herbivory and weather on the vital rates of two closely related cactus species. Ecol Evol. 2017;00:1–14.
https://doi.org/10.1002/ece3.3232

1 Model Selection Methods

Using the Akaike Information Criterion (AIC), we compared a number of models containing the full set of predictor variables (fixed effects) to test whether the inclusion of various random effects improved model fit (Burnham and Anderson 2007). We considered four factors in combination and alone: plant identity (unique to each plant), location, year, and location × year. We evaluated the fit of the models using likelihood ratio tests based on the asymptotic χ^2 distribution (Proc GLIMMIX, COVTEST CL [TYPE=ESTIMATED] option, SAS/STAT 13.2). Next, including the best random effect(s), we generated models that varied in their fixed effects and ranked them using conditional AIC (Vaida and Blanchard 2005; cAIC; Müller et al. 2013). We considered 2 and 3 way interactions.

1.1 Models of *Opuntia humifusa* RGR

We chose to use the random effects "Location X Year" and "PlantID" in our fixed effect models (models that varied their inlusion of fixed effects); the full model with these random effects had a very small ΔAIC (the difference between the model with the lowest AIC and a given model's AIC) and both random effects significantly improved model fit when included as the only random effect in the full model.

Table S1: Rankings of Models with Different Random Effects. β_0 is the intercept; C_t is plant size at time t, standardized; the Native Bug, Native Moth, and Native Scale columns represent the presence/absence of that insect species at time t; P1 and P2 are the first and second axes resulting from the Precipitation Principal Components Analysis (PCA); T1 is the first axis resulting from the Temperature PCA; ΔAIC is the difference between the model with the lowest AIC and a given model's AIC; the model likelihood, Model $\mathcal{L} = e^{\left(-\frac{1}{2}\Delta\text{AIC}\right)}$; and $P(\text{Model}) = \mathcal{L}_i / \sum_{i=1}^{n} \mathcal{L}_i$ (Burnham and Anderson 2007).

Random Effects	β_0	C_t	Native Bug	Native Moth	Native Scale	P1	P2	T1	ΔAIC	Model $\mathcal{L}$	P(Model)
Location X Year	0.23	-0.54	-0.03	-0.18	0	-0.17	0.15	0.2	0	1	0.61
Location X Year, Plant ID (Intercept)	0.23	-0.6	-0.03	-0.19	0	-0.17	0.15	0.2	0.92	0.63	0.39
Location	0.33	-0.66	-0.09	-0.29	-0.12	-0.16	0.04	0.08	63.3	0	0
Location, Plant ID (Intercept)	0.33	-0.77	-0.1	-0.29	-0.13	-0.16	0.04	0.08	63.72	0	0
Year, Plant ID (Intercept)	0.25	-0.46	-0.06	-0.22	-0.07	-0.03	0.08	0.27	82	0	0
Year	0.24	-0.25	-0.03	-0.18	-0.04	0	0.08	0.31	91.28	0	0
Plant ID (Intercept)	0.32	-0.61	-0.12	-0.22	-0.07	-0.09	0.05	0.13	137.1	0	0

In our models varying in their fixed effects, we including "Location (Year)" and "PlantID" as random effects. We favored Model 2 because it is within 2 ΔcAIC units of the best model and has fewer parameters. Estimates and confidence intervals (in brackets) are given for each parameter included in each model.

Table S2: Rankings of Models with Different Fixed Effects. The P x T and Insect x Weather columns indicate interactions among those variables. The DF ("Degrees of Freedom") column formula is Number of Fixed Effect Parameters + Number of Random Effect Parameters = Total Number of Parameters. ΔcAIC is the difference between the model with the lowest cAIC and a given model's cAIC. $P(\text{Model}) = \mathcal{L}_i / \sum_{i=1}^{n} \mathcal{L}_i$ (Burnham and Anderson 2007).

Model	β_0	C_t	Bug	Scale	Native Moth	Native Insects	P	T	P x T	Insect x Weather	Location x Year	PlantID	DF	ΔcAIC	P(Model)
1	0.22 [0.017, 0.424]	-0.606 [-0.812, -0.399]			-0.187 [-0.379, 0.006]		P1 = -0.169 [-0.264, -0.074], P2 = 0.152 [0.074, 0.23]	T1 = 0.198 [0.115, 0.281]			0.25	0.02	7 + 133 = 140	0	0.38
2	0.189 [-0.01, 0.388]	-0.632 [-0.838, -0.426]					P1 = -0.172 [-0.267, -0.077], P2 = 0.153 [0.075, 0.232]	T1 = 0.196 [0.112, 0.278]			0.24	0.02	5 + 133 = 138	0.72	0.26
3	0.227 [0.009, 0.445]	-0.614 [-0.824, -0.404]				-0.066 [-0.214, 0.083]	P1 = -0.172 [-0.267, -0.078], P2 = 0.152 [0.074, 0.23]	T1 = 0.192 [0.109, 0.275]			0.24	0.02	7 + 133 = 140	1.72	0.16
4	0.224 [0.01, 0.438]	-0.606 [-0.821, -0.391]	-0.058 [-0.206, 0.089]	0.005 [-0.166, 0.176]	-0.191 [-0.383, 0.002]		P1 = -0.206 [-0.306, -0.105], P2 = 0.132 [0.044, 0.221]	T1 = 0.201 [0.118, 0.283]		Native Bug x P = 0.168 [0.027, 0.31]	0.24	0.02	15 + 133 = 148	3.48	0.07
5	0.198 [-0.005, 0.401]	-0.626 [-0.834, -0.419]	-0.033 [-0.179, 0.113]				P1 = -0.17 [-0.265, -0.075], P2 = 0.152 [0.074, 0.231]	T1 = 0.195 [0.112, 0.278]			0.24	0.02	7 + 133 = 140	4.14	0.05

Model	β_0	C_t	Bug	Scale	Native Moth	Native Insects	P	T	P x T	Insect x Weather	Location x Year	PlantID	DF	ΔcAIC	P(Model)
6	0.191 [-0.018, 0.4]	-0.631 [-0.839, -0.424]		-0.005 [-0.176, 0.167]			P1 = -0.172 [-0.268, -0.077], P2 = 0.153 [0.075, 0.232]	T1 = 0.195 [0.112, 0.278]			0.24	0.02	7 + 133 = 140	4.66	0.04
7	0.19 [-0.029, 0.409]	-0.599 [-0.808, -0.389]	-0.026 [-0.171, 0.119]	0.007 [-0.165, 0.178]	-0.177 [-0.369, 0.016]		P1 = -0.185 [-0.285, -0.085], P2 = 0.101 [0.006, 0.196]	T1 = 0.198 [0.114, 0.282]	T1 x P1 = -0.088 [-0.187, 0.012], T1 x P2 = -0.08 [-0.16, 0.001]		0.24	0.02	13 + 133 = 146	5.97	0.02
8	0.22 [0.004, 0.436]	-0.601 [-0.811, -0.391]	-0.029 [-0.174, 0.116]	0.008 [-0.164, 0.179]	-0.185 [-0.378, 0.007]		P1 = -0.159 [-0.255, -0.063], P2 = 0.107 [0.012, 0.202]	T1 = 0.206 [0.123, 0.29]	T1 x P2 = -0.065 [-0.144, 0.014]		0.24	0.02	12 + 133 = 145	7.17	0.01
9	0.209 [-0.01, 0.429]	-0.597 [-0.807, -0.387]	-0.029 [-0.175, 0.116]	-0.007 [-0.178, 0.164]	-0.18 [-0.373, 0.013]		P1 = -0.189 [-0.29, -0.088], P2 = 0.155 [0.076, 0.233]	T1 = 0.189 [0.106, 0.273]	T1 x P1 = -0.067 [-0.165, 0.03]		0.25	0.02	12 + 133 = 145	7.35	0.01
10	0.23 [0.014, 0.447]	-0.599 [-0.809, -0.389]	-0.031 [-0.177, 0.114]	-0.004 [-0.175, 0.167]	-0.186 [-0.379, 0.007]		P1 = -0.168 [-0.263, -0.072], P2 = 0.151 [0.073, 0.23]	T1 = 0.197 [0.114, 0.28]			0.25	0.02	11 + 133 = 144	7.48	0.01
11	0.233 [0.015, 0.45]	-0.604 [-0.815, -0.393]	-0.03 [-0.176, 0.115]	-0.006 [-0.177, 0.166]	-0.18 [-0.374, 0.013]		P1 = -0.17 [-0.266, -0.074], P2 = 0.152 [0.074, 0.23]	T1 = 0.217 [0.12, 0.313]		Native Scale x T = -0.052 [-0.184, 0.079]	0.25	0.02	13 + 133 = 146	9.24	0

Model	β_0	C_t	Bug	Scale	Native Moth	Native Insects	P	T	P x T	Insect x Weather	Location x Year	PlantID	DF	ΔcAIC	P(Model)
12	0.23 [0.014, 0.446]	-0.599 [-0.81, -0.389]	-0.035 [-0.182, 0.112]	-0.003 [-0.174, 0.168]	-0.185 [-0.378, 0.007]		P1 = -0.167 [-0.262, -0.071], P2 = 0.151 [0.073, 0.23]	T1 = 0.205 [0.113, 0.296]		Native Bug x T = -0.027 [-0.162, 0.108]	0.25	0.02	13 + 133 = 146	11.27	0
13	0.23 [0.014, 0.447]	-0.598 [-0.808, -0.388]	-0.031 [-0.177, 0.114]	-0.007 [-0.179, 0.165]	-0.19 [-0.384, 0.004]		P1 = -0.166 [-0.262, -0.07], P2 = 0.151 [0.073, 0.229]	T1 = 0.194 [0.109, 0.279]		Native Moth x T = 0.031 [-0.15, 0.212]	0.25	0.02	13 + 133 = 146	11.37	0
14	0.228 [0.01, 0.446]	-0.6 [-0.81, -0.39]	-0.03 [-0.176, 0.115]	-0.024 [-0.199, 0.15]	-0.181 [-0.374, 0.012]		P1 = -0.195 [-0.314, -0.076], P2 = 0.13 [0.044, 0.218]	T1 = 0.192 [0.108, 0.275]		Native Scale x P = 0.048 [-0.087, 0.183]	0.25	0.02	15 + 133 = 148	13.81	0
15	0.232 [0.014, 0.449]	-0.599 [-0.809, -0.389]	-0.034 [-0.18, 0.112]	-0.002 [-0.174, 0.17]	-0.184 [-0.378, 0.009]		P1 = -0.177 [-0.28, -0.073], P2 = 0.146 [0.066, 0.225]	T1 = 0.193 [0.11, 0.277]		Native Moth x P = 0.017 [-0.144, 0.177]	0.25	0.02	15 + 133 = 148	14.42	0
16	0.203 [-0.009, 0.415]	-0.648 [-0.86, -0.435]					P1 = -0.305 [-0.384, -0.227], P2 = 0.149 [0.07, 0.228]				0.28	0.02	4 + 133 = 137	17.31	0
17	0.164 [-0.016, 0.344]	-0.631 [-0.834, -0.428]						T1 = 0.275 [0.207, 0.343]			0.19	0.01	3 + 133 = 136	38.94	0
18	0.247 [0.049, 0.446]	-0.631 [-0.854, -0.409]				-0.123 [-0.277, 0.031]					0.18	0.01	4 + 133 = 137	104.3	0

Model	β_0	C_t	Bug	Scale	Native Moth	Native Insects	P	T	P x T	Insect x Weather	Location x Year	PlantID	DF	ΔcAIC	P(Model)
19	0.208 [0.026, 0.389]	-0.637 [-0.855, -0.419]			-0.185 [-0.386, 0.017]						0.19	0.01	4 + 133 = 137	107.7	0
20	0.213 [0.034, 0.391]	-0.637 [-0.856, -0.418]	-0.132 [-0.283, 0.02]								0.17	0.01	4 + 133 = 137	107.7	0
21	0.177 [0.002, 0.351]	-0.66 [-0.877, -0.444]									0.18	0.01	2 + 133 = 135	109.8	0
22	0.225 [0.03, 0.421]	-0.621 [-0.842, -0.4]	-0.13 [-0.281, 0.021]	0.051 [-0.125, 0.226]	-0.183 [-0.384, 0.018]						0.18	0.01	8 + 133 = 141	111.1	0
23	0.159 [-0.027, 0.345]	-0.668 [-0.885, -0.45]		0.051 [-0.125, 0.227]							0.18	0.01	4 + 133 = 137	114.5	0

1.2 Models of ***Opuntia stricta*** RGR

We chose to include the random effect "Location X Year" in our candidate fixed effects models.

Table S3: Rankings of Models with Different Random Effects. The Native Bug and Invasive Moth columns represent the presence/absence of that insect species at time t.

Random Effects	β_0	C_t	Native Bug	Invasive Moth	P1	P2	T1	ΔAIC	Model $\mathcal{L}$	P(Model)
Location X Year	0.33	-0.25	-0.21	-0.41	-0.08	0.2	0.3	0	1	0.93
Year	0.31	-0.28	-0.18	-0.41	-0.01	0.13	0.33	5.22	0.07	0.07
Location	0.39	-0.24	-0.26	-0.37	-0.1	0.1	0.21	13.94	0	0

1.3 Candidate Fixed Effects Models

Next, we generated random effects models that varied in their fixed effects and ranked them using cAIC. We considered 2 and 3 way interactions. While Models 2 and 3 are within 2 Δ cAIC units of the best model, we favor Model 1 because it has fewer parameters than Model 2 and an equal number of parameters as Model 3.

Table S4: Rankings of Models with Different Fixed Effects.

Model	β_0	C_t	Native Bug	Invasive Moth	Native Insects	P	T	P x T	Insect x Weather	Location X Year	DF	ΔcAIC	P(Model)
1	0.361 [0.153, 0.57]	-0.25 [-0.439, -0.06]	-0.201 [-0.398, -0.004]	-0.4 [-0.627, -0.172]		P1 = -0.02 [-0.135, 0.096], P2 = 0.187 [0.083, 0.291]	T1 = 0.348 [0.239, 0.457]	T1 x P1 = 0.201 [0.059, 0.343]		0.09475	10 + 18 = 28	0	0.35
2	0.352 [0.142, 0.562]	-0.246 [-0.435, -0.057]	-0.205 [-0.402, -0.008]	-0.4 [-0.627, -0.173]		P1 = -0.019 [-0.135, 0.097], P2 = 0.153 [0.034, 0.272]	T1 = 0.344 [0.234, 0.453]	T1 x P1 = 0.176 [0.028, 0.324], T1 x P2 = -0.062 [-0.172, 0.049]		0.09577	11 + 18 = 29	0.57	0.27
3	0.322 [0.103, 0.54]	-0.245 [-0.434, -0.055]	-0.214 [-0.411, -0.016]	-0.407 [-0.635, -0.178]		P1 = -0.063 [-0.174, 0.049], P2 = 0.142 [0.022, 0.263]	T1 = 0.302 [0.198, 0.405]	T1 x P2 = -0.103 [-0.21, 0.005]		0.1123	10 + 18 = 28	1.4	0.17
4	0.332 [0.116, 0.548]	-0.252 [-0.442, -0.061]	-0.209 [-0.407, -0.01]	-0.408 [-0.638, -0.179]		P1 = -0.076 [-0.187, 0.036], P2 = 0.2 [0.092, 0.307]	T1 = 0.298 [0.195, 0.401]			0.1081	9 + 18 = 27	3.67	0.06
5	0.26 [0.055, 0.464]	-0.284 [-0.473, -0.095]		-0.423 [-0.653, -0.193]		P1 = -0.087 [-0.198, 0.024], P2 = 0.219 [0.113, 0.326]	T1 = 0.313 [0.21, 0.415]			0.1103	7 + 18 = 25	3.74	0.05
6	0.332 [0.112, 0.552]	-0.246 [-0.436, -0.056]	-0.203 [-0.401, -0.004]	-0.412 [-0.641, -0.184]		P1 = -0.071 [-0.184, 0.041], P2 = 0.204 [0.096, 0.311]	T1 = 0.257 [0.135, 0.378]		Native Bug x T = 0.115 [-0.064, 0.294]	0.1156	11 + 18 = 29	4.88	0.03
7	0.332 [0.112, 0.552]	-0.242 [-0.432, -0.052]	-0.194 [-0.392, 0.005]	-0.413 [-0.642, -0.184]		P1 = -0.069 [-0.18, 0.043], P2 = 0.202 [0.096, 0.309]	T1 = 0.265 [0.13, 0.4]		Invasive Moth x Native Bug x T	0.116	13 + 18 = 31	6.07	0.02
8	0.324 [0.11, 0.539]	-0.254 [-0.446, -0.063]		-0.407 [-0.636, -0.177]	-0.17 [-0.366, 0.026]	P1 = -0.078 [-0.189, 0.033], P2 = 0.202 [0.093, 0.31]	T1 = 0.299 [0.196, 0.402]			0.1014	9 + 18 = 27	6.15	0.02
9	0.333 [0.117, 0.548]	-0.247 [-0.438, -0.057]	-0.208 [-0.407, -0.01]	-0.403 [-0.632, -0.173]		P1 = -0.074 [-0.186, 0.037], P2 = 0.197 [0.09, 0.305]	T1 = 0.327 [0.211, 0.443]		Invasive Moth x T = -0.099 [-0.284, 0.085]	0.1073	11 + 18 = 29	6.64	0.01

Model	β_0	C_t	Native Bug	Invasive Moth	Native Insects	P	T	P x T	Insect x Weather	Location X Year	DF	ΔcAIC	P(Model)
10	0.26 [0.056, 0.464]	-0.28 [-0.468, -0.091]		-0.417 [-0.647, -0.188]		P1 = -0.086 [-0.197, 0.025], P2 = 0.217 [0.111, 0.323]	T1 = 0.342 [0.226, 0.458]		Invasive Moth x T = -0.1 [-0.285, 0.085]	0.1094	9 + 18 = 27	6.71	0.01
11	0.333 [0.118, 0.549]	-0.253 [-0.444, -0.063]	-0.204 [-0.404, -0.004]	-0.402 [-0.632, -0.173]		P1 = -0.024 [-0.17, 0.123], P2 = 0.217 [0.09, 0.344]	T1 = 0.298 [0.195, 0.401]		Native Bug x P = -0.095 [-0.277, 0.087]	0.1072	13 + 18 = 31	10.62	0
12	0.35 [0.13, 0.571]	-0.254 [-0.444, -0.063]	-0.261 [-0.491, -0.031]	-0.488 [-0.779, -0.198]		P1 = -0.078 [-0.19, 0.034], P2 = 0.198 [0.09, 0.305]	T1 = 0.297 [0.194, 0.4]			0.1092	13 + 18 = 31	10.69	0
13	0.236 [0.023, 0.448]	-0.376 [-0.556, -0.197]	-0.231 [-0.431, -0.03]			P1 = -0.079 [-0.192, 0.035], P2 = 0.192 [0.082, 0.301]	T1 = 0.279 [0.175, 0.382]			0.1141	7 + 18 = 25	11.29	0
14	0.334 [0.121, 0.548]	-0.253 [-0.443, -0.062]	-0.208 [-0.407, -0.01]	-0.398 [-0.629, -0.167]		P1 = -0.081 [-0.204, 0.042], P2 = 0.211 [0.099, 0.324]	T1 = 0.296 [0.193, 0.399]		Invasive Moth x P = 0.021 [-0.168, 0.209]	0.1037	13 + 18 = 31	11.78	0
15	0.262 [0.06, 0.463]	-0.285 [-0.474, -0.097]		-0.412 [-0.644, -0.181]		P1 = -0.091 [-0.214, 0.032], P2 = 0.232 [0.12, 0.343]	T1 = 0.31 [0.208, 0.413]		Invasive Moth x P = 0.014 [-0.175, 0.203]	0.1057	11 + 18 = 29	11.84	0
16	0.151 [-0.048, 0.351]	-0.418 [-0.594, -0.241]				P1 = -0.091 [-0.205, 0.022], P2 = 0.213 [0.105, 0.321]	T1 = 0.294 [0.191, 0.398]			0.1168	5 + 18 = 23	12.14	0
17	0.235 [0.019, 0.452]	-0.372 [-0.552, -0.193]	-0.225 [-0.426, -0.025]			P1 = -0.075 [-0.189, 0.039], P2 = 0.195 [0.086, 0.304]	T1 = 0.24 [0.118, 0.363]		Native Bug x T = 0.106 [-0.075, 0.287]	0.1208	9 + 18 = 27	12.94	0
18	0.335 [0.122, 0.548]	-0.27 [-0.461, -0.079]	-0.228 [-0.433, -0.023]	-0.342 [-0.584, -0.1]		P1 = -0.066 [-0.225, 0.093], P2 = 0.224 [0.091, 0.357]	T1 = 0.293 [0.19, 0.396]		Invasive Moth x Native Bug x P	0.1026	17 + 18 = 35	16.62	0

Model	β_0	C_t	Native Bug	Invasive Moth	Native Insects	P	T	P x T	Insect x Weather	Location X Year	DF	ΔcAIC	P(Model)
19	0.239 [0.027, 0.451]	-0.377 [-0.556, -0.198]	-0.224 [-0.426, -0.022]			P1 = -0.018 [-0.167, 0.13], P2 = 0.211 [0.082, 0.34]	T1 = 0.279 [0.176, 0.382]		Native Bug x P = -0.11 [-0.294, 0.074]	0.1134	11 + 18 = 29	17.84	0
20	0.151 [-0.004, 0.305]	-0.412 [-0.592, -0.231]					T1 = 0.3 [0.204, 0.395]			0.0515	3 + 18 = 21	38.34	0
21	0.175 [-0.04, 0.389]	-0.434 [-0.617, -0.252]				P1 = -0.239 [-0.354, -0.123], P2 = 0.204 [0.092, 0.317]				0.1397	4 + 18 = 22	38.73	0
22	0.302 [0.143, 0.461]	-0.368 [-0.557, -0.179]	-0.375 [-0.581, -0.168]							0.0317	4 + 18 = 22	71.83	0
23	0.382 [0.22, 0.544]	-0.268 [-0.47, -0.067]	-0.355 [-0.562, -0.148]	-0.319 [-0.556, -0.082]						0.02118	6 + 18 = 24	73.18	0
24	0.4 [0.231, 0.57]	-0.271 [-0.473, -0.07]	-0.412 [-0.654, -0.17]	-0.402 [-0.703, -0.102]						0.02348	10 + 18 = 28	79.32	0
25	0.387 [0.233, 0.541]	-0.264 [-0.467, -0.062]		-0.316 [-0.551, -0.08]	-0.326 [-0.524, -0.127]					0.009816	6 + 18 = 24	80.37	0
26	0.404 [0.242, 0.566]	-0.27 [-0.472, -0.067]		-0.402 [-0.711, -0.092]	-0.378 [-0.611, -0.144]					0.01217	10 + 18 = 28	86.21	0
27	0.169 [0.046, 0.292]	-0.433 [-0.621, -0.246]								0.01461	2 + 18 = 20	88.58	0
28	0.27 [0.138, 0.402]	-0.316 [-0.519, -0.112]		-0.35 [-0.586, -0.114]						0.001814	4 + 18 = 22	92.79	0

2 References

Burnham KP, Anderson DR (2007) Model selection and multimodel inference: A practical information- theoretic approach. Springer Science & Business Media, New York, USA M{"u}ller S, Scealy JL, Welsh AH (2013) Model selection in linear mixed models. Stat Sci 28:135-167. Vaida F, Blanchard S (2005) Conditional Akaike information for mixed-effects models. Biometrika 92:351-370.
