# Supplementary material for: The influence of herbivory and weather on the vital rates of two closely related cactus species
Source: Ecol Evol. 2017 Jul 31;7(17):6996–7009. doi: 10.1002/ece3.3232 (PMC5587481; doi:10.1002/ece3.3232)
Supplement: Supplementary file 3 [file ECE3-7-6996-s003.pdf]

Appendix S3: Selection of Models Predicting Fruit Presence and Abundance

for the article:
Sauby KE, Kilmer J, Christman MC, Holt RD, Marsico TD. The influence of herbivory and weather on the vital rates of two closely related cactus species. Ecol Evol. 2017;00:1–14.
https://doi.org/10.1002/ece3.3232

1 Construction of Zero-Inflated Random Effects Poisson Models

For each of the two cactus species, we ran zero-inflated Poisson models first to determine which parameters were significant (proc GENMOD, SAS/STAT 13.2). We removed the non-significant parameters and we reran the zero-inflated Poisson model to obtain parameter estimates to use as starting values for the zero-inflated Poisson model with random effects. We then ran generalized linear mixed models to obtain covariance (random effect) parameter estimates, which we used as starting values in the zero-inflated Poisson models with random effects. We used the set of significant parameters as fixed effects and ran a model per each covariance parameter.

We next ran zero-inflated Poisson models with random effects to determine which random effects explained the most variance in the data (proc NLMIXED, SAS/STAT 13.2). We considered four random effects: Fecundity Year, Location, Location (Fecundity Year), and Plant ID. We used AIC (-2 Log-Likelihood $+2p$, Burnham and Anderson 2007) to rank the candidate models with respect to the random effects, keeping the same set of fixed effects for all models.

2 *O. humifusa*

2.1 Comparison of Zero-Inflated Random Effects Poisson Models

The best model included Plant ID as a random effect in the count component.

Table S1: Comparison of Random Effect Models. γ is the random effect; β_0 is the intercept; C_t is plant size at time t, standardized; Native Bug is the presence/absence of the Native Bug at time t; ΔAIC is the difference between the model with the lowest AIC and a given model's AIC; the model likelihood, Model $\mathcal{L} = e^{\left(-\frac{1}{2}\Delta\text{AIC}\right)}$; and P(Model) $= \mathcal{L}_i / \sum_{i=1}^{n} \mathcal{L}_i$ (Burnham and Anderson 2007).

Model	Vital Rate	γ	β_0	C_t	Native Bug	Precipitation	Temperature	ΔAIC	Model $\mathcal{L}$	P(Model)
**1**	**Zero**		**0.32 [-0.21, 0.84]**	**-1.22 [-2.18, -0.27]**	**-0.78 [-1.46, -0.1]**	**P1 (Spring/Summer) = -0.62 [-0.98, -0.27]**	**Mean Max. Temp (Spring/Summer) = -2.17 [-3.01, -1.33]**	**0**	**1**	**1**
**1**	**Count**	**Plant ID = 0.3 [0.12, 0.48]**	**1.03 [0.76, 1.3]**	**2.48 [2.22, 2.74]**	**-0.16 [-0.26, -0.07]**	**P1 (Fall/Winter) = 0.13 [0.08, 0.18]**	**Mean Degree Day (Spring/Summer) = -0.97 [-1.17, -0.76], Mean Max. Temp (Spring/Summer) = 0.67 [0.42, 0.91], T1 (Fall/Winter) = 0.24 [0.16, 0.32]**			
2	Zero	Plant ID = 2.95 [0.07, 5.83]	0.85 [0.1, 1.6]	-2.1 [-3.57, -0.63]	-1.14 [-2.02, -0.26]	P1 (Spring/Summer) = -0.86 [-1.32, -0.4]	Mean Max. Temp (Spring/Summer) = -3.29 [-4.76, -1.83]	469.5	0	0
2	Count		1.42 [1.29, 1.54]	2.03 [1.91, 2.15]	-0.19 [-0.27, -0.12]	P1 (Fall/Winter) = 0.17 [0.14, 0.2]	Mean Degree Day (Spring/Summer) = -0.98 [-1.1, -0.86], Mean Max. Temp (Spring/Summer) = 0.79 [0.66, 0.92], T1 (Fall/Winter) = 0.1 [0.04, 0.17]			
3	Zero	Location = 0.29 [-0.46, 1.04]	0.59 [-0.38, 1.55]	-1.65 [-3.04, -0.26]	-0.82 [-1.73, 0.1]	P1 (Spring/Summer) = -0.56 [-1.08, -0.04]	Mean Max. Temp (Spring/Summer) = -2.27 [-3.91, -0.63]	482.9	0	0

Model	Vital Rate	γ	β_0	C_t	Native Bug	Precipitation	Temperature	ΔAIC	Model $\mathcal{L}$	P(Model)
3	Count		1.42 [1.24, 1.59]	2.03 [1.87, 2.2]	-0.19 [-0.3, -0.08]	P1 (Fall/Winter) = 0.17 [0.12, 0.22]	Mean Degree Day (Spring/Summer) = -0.98 [-1.14, -0.82], Mean Max. Temp (Spring/Summer) = 0.79 [0.61, 0.97], T1 (Fall/Winter) = 0.1 [0.01, 0.19]			
4	Zero		0.55 [0.08, 1.02]	-1.55 [-2.41, -0.69]	-0.76 [-1.38, -0.14]	P1 (Spring/Summer) = -0.65 [-0.97, -0.34]	Mean Max. Temp (Spring/Summer) = -2.25 [-3.05, -1.46]	486.6	0	0
4	Count		1.41 [1.29, 1.54]	2.03 [1.91, 2.15]	-0.19 [-0.27, -0.12]	P1 (Fall/Winter) = 0.17 [0.14, 0.21]	Mean Degree Day (Spring/Summer) = -0.98 [-1.1, -0.87], Mean Max. Temp (Spring/Summer) = 0.79 [0.67, 0.92], T1 (Fall/Winter) = 0.1 [0.04, 0.16]			
5	Zero	Location = 0.29 [-0.58, 1.16]	0.59 [-0.52, 1.69]	-1.65 [-3.24, -0.06]	-0.82 [-1.87, 0.24]	P1 (Spring/Summer) = -0.56 [-1.16, 0.03]	Mean Max. Temp (Spring/Summer) = -2.27 [-4.15, -0.39]	486.9	0	0
5	Count	Location = 0 [NA, NA]	1.42 [1.22, 1.62]	2.03 [1.84, 2.22]	-0.19 [-0.32, -0.07]	P1 (Fall/Winter) = 0.17 [0.12, 0.23]	Mean Degree Day (Spring/Summer) = -0.98 [-1.17, -0.79], Mean Max. Temp (Spring/Summer) = 0.79 [0.58, 1], T1 (Fall/Winter) = 0.1 [0, 0.2]			
6	Zero	Location (Fecundity Year) = 0.29 [-0.27, 0.84], Fecundity Year = 0 [NA, NA]	0.61 [0.02, 1.21]	-1.62 [-2.62, -0.62]	-0.84 [-1.56, -0.12]	P1 (Spring/Summer) = -0.69 [-1.13, -0.24]	Mean Max. Temp (Spring/Summer) = -2.34 [-3.39, -1.29]	488.2	0	0
6	Count		1.41 [1.27, 1.55]	2.03 [1.9, 2.16]	-0.19 [-0.28, -0.11]	P1 (Fall/Winter) = 0.17 [0.13, 0.21]	Mean Degree Day (Spring/Summer) = -0.98 [-1.11, -0.86], Mean Max. Temp (Spring/Summer) = 0.79 [0.65, 0.94], T1 (Fall/Winter) = 0.1 [0.03, 0.17]			

Model	Vital Rate	γ	β_0	C_t	Native Bug	Precipitation	Temperature	ΔAIC	Model $\mathcal{L}$	P(Model)
7	Zero	Location (Fecundity Year) = 0.26 [-0.26, 0.78]	0.55 [-0.03, 1.13]	-1.55 [-2.53, -0.57]	-0.76 [-1.47, -0.05]	P1 (Spring/Summer) = -0.66 [-1.09, -0.23]	Mean Max. Temp (Spring/Summer) = -2.26 [-3.28, -1.24]	488.3	0	0
7	Count	Fecundity Year = 0 [NA, NA]	1.41 [1.27, 1.55]	2.03 [1.91, 2.16]	-0.2 [-0.28, -0.11]	P1 (Fall/Winter) = 0.17 [0.12, 0.23]	Mean Degree Day (Spring/Summer) = -0.99 [-1.15, -0.82], Mean Max. Temp (Spring/Summer) = 0.8 [0.62, 0.97], T1 (Fall/Winter) = 0.1 [-0.01, 0.21]			
8	Zero	Fecundity Year = 0 [NA, NA]	0.55 [-0.21, 1.31]	-1.55 [-2.93, -0.16]	-0.76 [-1.76, 0.25]	P1 (Spring/Summer) = -0.65 [-1.16, -0.14]	Mean Max. Temp (Spring/Summer) = -2.25 [-3.54, -0.97]	488.6	0	0
8	Count		1.41 [1.21, 1.61]	2.03 [1.84, 2.22]	-0.19 [-0.32, -0.07]	P1 (Fall/Winter) = 0.17 [0.12, 0.23]	Mean Degree Day (Spring/Summer) = -0.98 [-1.17, -0.79], Mean Max. Temp (Spring/Summer) = 0.79 [0.59, 1], T1 (Fall/Winter) = 0.1 [0, 0.2]			
9	Zero		0.55 [-0.21, 1.31]	-1.55 [-2.93, -0.16]	-0.76 [-1.76, 0.25]	P1 (Spring/Summer) = -0.65 [-1.16, -0.14]	Mean Max. Temp (Spring/Summer) = -2.25 [-3.54, -0.97]	488.6	0	0
9	Count	Fecundity Year = 0 [NA, NA]	1.41 [1.21, 1.61]	2.03 [1.84, 2.22]	-0.19 [-0.32, -0.07]	P1 (Fall/Winter) = 0.17 [0.12, 0.23]	Mean Degree Day (Spring/Summer) = -0.98 [-1.17, -0.79], Mean Max. Temp (Spring/Summer) = 0.79 [0.59, 1], T1 (Fall/Winter) = 0.1 [0, 0.2]			
10	Zero		0.55 [-0.11, 1.21]	-1.55 [-2.76, -0.34]	-0.76 [-1.63, 0.12]	P1 (Spring/Summer) = -0.65 [-1.09, -0.2]	Mean Max. Temp (Spring/Summer) = -2.25 [-3.37, -1.13]	488.6	0	0

Model	Vital Rate	γ	β_0	C_t	Native Bug	Precipitation	Temperature	ΔAIC	Model $\mathcal{L}$	P(Model)
10	Count	Location = 0 [NA, NA]	1.42 [1.24, 1.59]	2.03 [1.87, 2.2]	-0.19 [-0.3, -0.08]	P1 (Fall/Winter) = 0.17 [0.12, 0.22]	Mean Degree Day (Spring/Summer) = -0.98 [-1.15, -0.82], Mean Max. Temp (Spring/Summer) = 0.79 [0.61, 0.97], T1 (Fall/Winter) = 0.1 [0.01, 0.19]			
11	Zero	Fecundity Year = 0 [NA, NA]	0.55 [0.05, 1.05]	-1.55 [-2.46, -0.63]	-0.76 [-1.42, -0.09]	P1 (Spring/Summer) = -0.65 [-0.99, -0.31]	Mean Max. Temp (Spring/Summer) = -2.25 [-3.1, -1.4]	490.6	0	0
11	Count	Location (Fecundity Year) = 0 [NA, NA]	1.41 [1.28, 1.55]	2.03 [1.9, 2.16]	-0.19 [-0.28, -0.11]	P1 (Fall/Winter) = 0.17 [0.12, 0.23]	Mean Degree Day (Spring/Summer) = -0.98 [-1.15, -0.82], Mean Max. Temp (Spring/Summer) = 0.79 [0.62, 0.96], T1 (Fall/Winter) = 0.1 [0, 0.2]			
12	Zero		0.55 [0.05, 1.05]	-1.55 [-2.46, -0.63]	-0.76 [-1.42, -0.09]	P1 (Spring/Summer) = -0.65 [-0.99, -0.31]	Mean Max. Temp (Spring/Summer) = -2.25 [-3.1, -1.41]	490.6	0	0
12	Count	Location (Fecundity Year) = 0 [NA, NA], Fecundity Year = 0 [NA, NA]	1.41 [1.27, 1.56]	2.03 [1.9, 2.16]	-0.19 [-0.28, -0.11]	P1 (Fall/Winter) = 0.17 [0.11, 0.24]	Mean Degree Day (Spring/Summer) = -0.98 [-1.18, -0.79], Mean Max. Temp (Spring/Summer) = 0.79 [0.6, 0.99], T1 (Fall/Winter) = 0.1 [-0.03, 0.23]			
13	Zero	Fecundity Year = 0 [NA, NA]	0.55 [-0.47, 1.57]	-1.55 [-3.42, 0.33]	-0.76 [-2.11, 0.6]	P1 (Spring/Summer) = -0.65 [-1.34, 0.04]	Mean Max. Temp (Spring/Summer) = -2.25 [-3.99, -0.52]	492.6	0	0
13	Count	Fecundity Year = 0 [NA, NA]	1.41 [1.14, 1.68]	2.03 [1.78, 2.29]	-0.19 [-0.36, -0.02]	P1 (Fall/Winter) = 0.17 [0.1, 0.25]	Mean Degree Day (Spring/Summer) = -0.98 [-1.24, -0.73], Mean Max. Temp (Spring/Summer) = 0.79 [0.51, 1.08], T1 (Fall/Winter) = 0.1 [-0.04, 0.24]			

2.2 Selection of Fixed Effect Interactions

Based on model selection of the random effects, we included Plant ID as a random effect in the count component of models. We next considered the inclusion of weather and insect interactions. There was a large amount of support for Model 1 based on the the Δ AIC between Models 1 and 2.

Table S2: Comparison of Models with Different Fixed Effects. β_0 is the intercept. C_t is plant size at time t, standardized. The Native Bug column represent the presence/absence of that insect species at time t. The P x T column represent interactions among Precipitation and Temperature variables. The DF ("Degrees of Freedom") column formula is Number of Fixed Effect Parameters + Number of Random Effect Parameters = Total Number of Parameters. ΔcAIC is the difference between the model with the lowest cAIC and a given model's cAIC. $P(\text{Model}) = \mathcal{L}_i / \sum_{i=1}^{n} \mathcal{L}_i$ (Burnham and Anderson 2007).

Model	Vital Rate	β_0	C_t	Native Bug	Precipitation	Temperature	P x T	Insect x Weather	DF	ΔcAIC	P(Model)
**9**	**Zero**	**0.2 [-0.36, 0.75]**	**-1.16 [-2.12, -0.2]**	**-0.6 [-1.29, 0.09]**	**P1 (Spring/Summer) = -0.63 [-0.98, -0.28]**	**Mean Max. Temp (Spring/Summer) = -2.21 [-3.05, -1.37]**			**14 + 96 = 110**	**0**	**1**
**9**	**Count**	**0.91 [0.64, 1.18]**	**2.57 [2.3, 2.83]**	**0.05 [-0.08, 0.18]**	**P1 (Fall/Winter) = 0.13 [0.08, 0.18]**	**Mean Degree Day (Spring/Summer) = -1.49 [-1.79, -1.19], Mean Max. Temp (Spring/Summer) = 0.55 [0.31, 0.8], T1 (Fall/Winter) = 0.22 [0.14, 0.3]**		**Native Bug x Mean Degree Day (Spring/Summer) = 0.68 [0.39, 0.96]**			
10	Zero	0.25 [-0.3, 0.79]	-1.19 [-2.15, -0.23]	-0.66 [-1.35, 0.03]	P1 (Spring/Summer) = -0.63 [-0.98, -0.29]	Mean Max. Temp (Spring/Summer) = -2.16 [-2.99, -1.33]			14 + 96 = 110	14.8	0
10	Count	0.93 [0.66, 1.2]	2.49 [2.24, 2.75]	0.02 [-0.15, 0.18]	P1 (Fall/Winter) = 0.13 [0.08, 0.18]	Mean Degree Day (Spring/Summer) = -0.93 [-1.13, -0.73], Mean Max. Temp (Spring/Summer) = 0.64 [0.4, 0.88], T1 (Fall/Winter) = 0.39 [0.26, 0.53]		Native Bug x T1 (Fall/Winter) = -0.2 [-0.34, -0.05]			

Model	Vital Rate	β_0	C_t	Native Bug	Precipitation	Temperature	P x T	Insect x Weather	DF	ΔcAIC	P(Model)
8	Zero	0.36 [-0.15, 0.88]	-1.23 [-2.18, -0.27]	-0.92 [-1.62, -0.21]	P1 (Spring/Summer) = -0.61 [-0.97, -0.25]	Mean Max. Temp (Spring/Summer) = -2.17 [-3.01, -1.32]			14 + 96 = 110	14.9	0
8	Count	1.15 [0.87, 1.44]	2.53 [2.27, 2.8]	-0.4 [-0.6, -0.2]	P1 (Fall/Winter) = 0.13 [0.07, 0.18]	Mean Degree Day (Spring/Summer) = -1.02 [-1.24, -0.81], Mean Max. Temp (Spring/Summer) = 0.44 [0.14, 0.74], T1 (Fall/Winter) = 0.26 [0.18, 0.34]		Native Bug x Mean Max. Temp (Spring/Summer) = 0.32 [0.08, 0.57]			
5	Zero	0.31 [-0.22, 0.84]	-1.24 [-2.2, -0.28]	-0.77 [-1.45, -0.08]	P1 (Spring/Summer) = -0.67 [-1.01, -0.32]	Mean Max. Temp (Spring/Summer) = -2.02 [-2.86, -1.17]			14 + 96 = 110	16.2	0
5	Count	0.96 [0.68, 1.25]	2.47 [2.21, 2.73]	-0.15 [-0.24, -0.05]	P1 (Fall/Winter) = 0 [-0.11, 0.12]	Mean Degree Day (Spring/Summer) = -0.75 [-1.02, -0.48], Mean Max. Temp (Spring/Summer) = 0.9 [0.58, 1.22], T1 (Fall/Winter) = 0.29 [0.2, 0.37]	P1 (Fall/Winter) x Mean Degree Day (Spring/Summer) = -0.53 [-0.98, -0.08]				
11	Zero	0.28 [-0.26, 0.82]	-1.22 [-2.18, -0.25]	-0.77 [-1.46, -0.07]	P1 (Spring/Summer) = -0.64 [-0.99, -0.29]	Mean Max. Temp (Spring/Summer) = -2.02 [-2.87, -1.18]			14 + 96 = 110	16.8	0

Model	Vital Rate	β_0	C_t	Native Bug	Precipitation	Temperature	P x T	Insect x Weather	DF	ΔcAIC	P(Model)
11	Count	1.02 [0.73, 1.3]	2.47 [2.21, 2.73]	-0.15 [-0.24, -0.05]	P1 (Fall/Winter) = 0.12 [0.07, 0.17]	Mean Degree Day (Spring/Summer) = -0.83 [-1.07, -0.6], Mean Max. Temp (Spring/Summer) = 0.8 [0.52, 1.09], T1 (Fall/Winter) = 0.37 [0.23, 0.51]	Mean Max. Temp (Spring/Summer) x T1 (Fall/Winter) = -0.21 [-0.4, -0.02]				
12	Zero	0.3 [-0.24, 0.84]	-1.2 [-2.17, -0.23]	-0.79 [-1.48, -0.1]	P1 (Spring/Summer) = -0.65 [-1, -0.29]	Mean Max. Temp (Spring/Summer) = -2.1 [-2.95, -1.25]			14 + 96 = 110	17.3	0
12	Count	0.94 [0.64, 1.24]	2.48 [2.22, 2.74]	-0.15 [-0.24, -0.06]	P1 (Fall/Winter) = 0.15 [0.1, 0.21]	Mean Degree Day (Spring/Summer) = -0.74 [-1.04, -0.45], Mean Max. Temp (Spring/Summer) = 0.85 [0.54, 1.16], T1 (Fall/Winter) = 0.2 [0.12, 0.29]	Mean Degree Day (Spring/Summer) x T1 (Fall/Winter) = -0.24 [-0.46, -0.01]				
4	Zero	0.29 [-0.23, 0.81]	-1.28 [-2.26, -0.3]	-0.69 [-1.38, 0]	P1 (Spring/Summer) = -0.31 [-0.77, 0.16]	Mean Max. Temp (Spring/Summer) = -2.22 [-3.08, -1.36]			14 + 96 = 110	18.1	0
4	Count	1.03 [0.77, 1.3]	2.49 [2.23, 2.75]	-0.16 [-0.25, -0.07]	P1 (Fall/Winter) = 0.13 [0.08, 0.18]	Mean Degree Day (Spring/Summer) = -0.96 [-1.16, -0.76], Mean Max. Temp (Spring/Summer) = 0.65 [0.41, 0.9], T1 (Fall/Winter) = 0.24 [0.16, 0.32]		Native Bug x P1 (Spring/Summer) = -0.68 [-1.39, 0.03]			

Model	Vital Rate	β_0	C_t	Native Bug	Precipitation	Temperature	P x T	Insect x Weather	DF	ΔcAIC	P(Model)
1	Zero	0.32 [-0.21, 0.84]	-1.22 [-2.18, -0.27]	-0.78 [-1.46, -0.1]	P1 (Spring/Summer) = -0.62 [-0.98, -0.27]	Mean Max. Temp (Spring/Summer) = -2.17 [-3.01, -1.33]			13 + 96 = 109	19.8	0
1	Count	1.03 [0.76, 1.3]	2.48 [2.22, 2.74]	-0.16 [-0.26, -0.07]	P1 (Fall/Winter) = 0.13 [0.08, 0.18]	Mean Degree Day (Spring/Summer) = -0.97 [-1.17, -0.76], Mean Max. Temp (Spring/Summer) = 0.67 [0.42, 0.91], T1 (Fall/Winter) = 0.24 [0.16, 0.32]					
2	Zero	0.31 [-0.21, 0.84]	-1.23 [-2.18, -0.28]	-0.76 [-1.44, -0.08]	P1 (Spring/Summer) = -0.61 [-0.96, -0.26]	Mean Max. Temp (Spring/Summer) = -2.16 [-2.99, -1.33]			14 + 96 = 110	21.1	0
2	Count	1.03 [0.76, 1.29]	2.5 [2.24, 2.76]	-0.16 [-0.26, -0.07]	P1 (Fall/Winter) = 0.18 [0.06, 0.3]	Mean Degree Day (Spring/Summer) = -0.91 [-1.15, -0.67], Mean Max. Temp (Spring/Summer) = 0.61 [0.34, 0.88], T1 (Fall/Winter) = 0.25 [0.17, 0.34]	P1 (Fall/Winter) x Mean Max. Temp (Spring/Summer) = -0.07 [-0.23, 0.09]				
6	Zero	0.3 [-0.23, 0.83]	-1.23 [-2.18, -0.27]	-0.78 [-1.46, -0.09]	P1 (Spring/Summer) = -0.62 [-0.97, -0.26]	Mean Max. Temp (Spring/Summer) = -2.13 [-2.97, -1.3]			14 + 96 = 110	21.5	0

Model	Vital Rate	β_0	C_t	Native Bug	Precipitation	Temperature	P x T	Insect x Weather	DF	ΔcAIC	P(Model)
6	Count	1.02 [0.74, 1.29]	2.47 [2.21, 2.73]	-0.16 [-0.25, -0.07]	P1 (Fall/Winter) = 0.15 [0.06, 0.24]	Mean Degree Day (Spring/Summer) = -0.94 [-1.16, -0.71], Mean Max. Temp (Spring/Summer) = 0.67 [0.42, 0.91], T1 (Fall/Winter) = 0.28 [0.12, 0.45]	P1 (Fall/Winter) x T1 (Fall/Winter) = -0.03 [-0.12, 0.07]				
3	Zero	0.31 [-0.22, 0.84]	-1.22 [-2.18, -0.26]	-0.75 [-1.45, -0.06]	P1 (Spring/Summer) = -0.63 [-0.98, -0.28]	Mean Max. Temp (Spring/Summer) = -2.43 [-3.86, -1.01]			14 + 96 = 110	21.6	0
3	Count	1.03 [0.76, 1.3]	2.48 [2.22, 2.74]	-0.16 [-0.26, -0.07]	P1 (Fall/Winter) = 0.13 [0.08, 0.18]	Mean Degree Day (Spring/Summer) = -0.97 [-1.17, -0.76], Mean Max. Temp (Spring/Summer) = 0.66 [0.42, 0.91], T1 (Fall/Winter) = 0.24 [0.16, 0.32]		Native Bug x Mean Max. Temp (Spring/Summer) = 0.4 [-1.3, 2.11]			
7	Zero	0.31 [-0.22, 0.84]	-1.23 [-2.19, -0.28]	-0.77 [-1.45, -0.09]	P1 (Spring/Summer) = -0.62 [-0.97, -0.27]	Mean Max. Temp (Spring/Summer) = -2.14 [-2.98, -1.31]			14 + 96 = 110	21.6	0
7	Count	1.02 [0.76, 1.29]	2.48 [2.22, 2.74]	-0.16 [-0.25, -0.06]	P1 (Fall/Winter) = 0.14 [0.08, 0.19]	Mean Degree Day (Spring/Summer) = -0.96 [-1.17, -0.76], Mean Max. Temp (Spring/Summer) = 0.66 [0.42, 0.91], T1 (Fall/Winter) = 0.25 [0.16, 0.33]		Native Bug x P1 (Fall/Winter) = -0.01 [-0.08, 0.05]			

3 *O. stricta*

3.1 Comparison of Zero-Inflated Random Effects Poisson Models

The best model included Fecundity Year as a random effect in the zero component and Location (Fecundity Year) in the count component. The second-best model was within two Δ AIC units of the best model, however in that model Fecundity Year had an estimate of zero.

Table S3: Comparison of Random Effect Models. γ is the random effect; β_0 is the intercept; C_t is plant size at time t, standardized; the Native Bug and Invasive Moth columns represent the presence/absence of that species at time t; ΔAIC is the difference between the model with the lowest AIC and a given model's AIC; the model likelihood, Model $\mathcal{L} = e^{\left(-\frac{1}{2}\Delta\text{AIC}\right)}$; and $P(\text{Model}) = \mathcal{L}_i / \sum_{i=1}^{n} \mathcal{L}_i$ (Burnham and Anderson 2007).

Model	Vital Rate	γ	β_0	C_t	Native Bug	Invasive Moth	ΔAIC	Model $\mathcal{L}$	P(Model)
**1**	**Zero**	**Fecundity Year = 0.59 [-1.71, 2.88]**	**3.37 [1.39, 5.35]**	**-4.46 [-6.98, -1.95]**			**0**	**1**	**0.6**
**1**	**Count**	**Location (Fecundity Year) = 1 [-0.06, 2.07]**	**0.15 [-0.3, 0.61]**	**5.16 [4.86, 5.47]**	**-0.93 [-1.27, -0.59]**	**-0.39 [-0.5, -0.27]**			
2	Zero		3.1 [1.61, 4.6]	-4.38 [-6.84, -1.92]			0.8	0.67	0.4
2	Count	Location (Fecundity Year) = 1.01 [-0.07, 2.08], Fecundity Year = 0 [NA, NA]	0.3 [-0.61, 1.21]	5.16 [4.86, 5.47]	-0.93 [-1.27, -0.59]	-0.39 [-0.5, -0.27]			
3	Zero		3.49 [2.22, 4.76]	-5 [-7.16, -2.83]			623	0	0
3	Count	Plant ID = 3.05 [-1.54, 7.64]	2.64 [1.15, 4.13]	1.93 [0.2, 3.66]	-0.43 [-0.62, -0.24]	-0.73 [-0.89, -0.57]			
4	Zero		3.21 [0.4, 6.03]	-4.53 [-9.23, 0.17]			882.3	0	0
4	Count	Location = 0.61 [-1.54, 2.76]	0.65 [-1.43, 2.74]	4.96 [4.36, 5.57]	-0.45 [-0.71, -0.19]	-0.77 [-0.91, -0.63]			
5	Zero	Location = 0.08 [-6.27, 6.42]	3.45 [-2.01, 8.91]	-4.85 [-37.99, 28.29]			887.4	0	0
5	Count	Location = 1.24 [-18.1, 20.59]	0.66 [-11.1, 12.42]	4.94 [4.53, 5.35]	-0.46 [-1.22, 0.3]	-0.77 [NA, NA]			
6	Zero		3.43 [1.37, 5.48]	-4.76 [-8.25, -1.26]			2213.9	0	0
6	Count	Fecundity Year = 0.35 [-0.44, 1.15]	1.44 [0.42, 2.46]	4.87 [4.51, 5.22]	-0.57 [-0.71, -0.43]	-1.4 [-1.54, -1.26]			
7	Zero	Location (Fecundity Year) = 0 [NA, NA]	3.53 [2.03, 5.03]	-4.98 [-7.56, -2.39]			2216	0	0
7	Count	Fecundity Year = 0.37 [-0.24, 0.97]	1.44 [0.71, 2.16]	4.86 [4.62, 5.11]	-0.57 [-0.67, -0.47]	-1.4 [-1.5, -1.3]			
8	Zero	Fecundity Year = 0.49 [-3.32, 4.31]	3.66 [0.05, 7.26]	-4.79 [-9.56, -0.02]			2217.2	0	0
8	Count	Fecundity Year = 0.35 [-0.72, 1.43]	1.44 [0.06, 2.82]	4.87 [4.39, 5.35]	-0.57 [-0.76, -0.38]	-1.4 [-1.58, -1.22]			
9	Zero		3.62 [2.39, 4.84]	-5.03 [-7.17, -2.9]			3042.2	0	0
9	Count		2.06 [1.88, 2.23]	3.91 [3.76, 4.06]	0.07 [0.01, 0.13]	-1.18 [-1.24, -1.12]			
10	Zero	Fecundity Year = 0.54 [-2.52, 3.6]	3.87 [1.22, 6.52]	-5.07 [-8.55, -1.6]			3043.4	0	0
10	Count		2.06 [1.78, 2.34]	3.91 [3.66, 4.16]	0.07 [-0.03, 0.17]	-1.18 [-1.28, -1.08]			

Model	Vital Rate	γ	β_0	C_t	Native Bug	Invasive Moth	ΔAIC	Model $\mathcal{L}$	P(Model)
11	Zero	Plant ID = 1.22 [-3.46, 5.91]	4.07 [1.92, 6.22]	-5.84 [-9.57, -2.12]			3043.7	0	0
11	Count		2.06 [1.88, 2.23]	3.91 [3.75, 4.07]	0.07 [0.01, 0.13]	-1.18 [-1.24, -1.12]			
12	Zero	Location = 0 [NA, NA]	3.62 [0.95, 6.29]	-5.03 [-9.69, -0.38]			3044.2	0	0
12	Count		2.06 [1.68, 2.43]	3.91 [3.58, 4.24]	0.07 [-0.06, 0.2]	-1.18 [-1.31, -1.04]			
13	Zero	Location (Fecundity Year) = 1.02 [NA, NA], Fecundity Year = 0.57 [-4.16, 5.3]	3.62 [1.89, 5.34]	-5.03 [-7.31, -2.76]			3048.7	0	0
13	Count		2.06 [NA, NA]	3.91 [NA, NA]	0.07 [0.01, 0.14]	-1.18 [-1.2, -1.16]			

4 Reference

Burnham KP, Anderson DR (2007) Model selection and multimodel inference: A practical information-theoretic approach. Springer Science & Business Media, New York, USA
